# Array-based polymer-phage biosensors for detection and differentiation of bacteria†

**DOI:** 10.1039/d5sd00069f

**Published:** 2025-07-02

**Authors:** Enkhlin Ochirbat, Junwhee Yang, Aritra Nath Chattopadhyay, Jungmi Park, Mingdi Jiang, Jan Paczesny, Vincent M. Rotello

**Affiliations:** a Institute of Physical Chemistry, Polish Academy of Sciences Kasprzaka 44/52 01224 Warsaw Poland jpaczesny@ichf.edu.pl; b Department of Chemistry, University of Massachusetts Amherst 710 N. Pleasant St Amherst MA 00103 USA rotello@umass.edu

## Abstract

Pathogenic bacteria, such as methicillin-resistant *Staphylococcus aureus* (MRSA), pose significant challenges to public health due to their resistance to conventional antibiotics. Early and accurate identification of bacterial species and discrimination of their strains is critical for guiding effective treatments and infection control. In this study, we develop a polymer-phage sensor platform that integrates polymer-based fluorescence sensing with phage-host specificity for bacterial identification. The sensor successfully differentiates three bacterial species (*S. aureus*, *E. coli*, and *B. subtilis*) and closely related strains of *S. aureus* (methicillin-sensitive *Staphylococcus aureus* (MSSA) and MRSA) with high classification accuracy (94–100%) and correct unknown identification rates (94–100%) under optimized conditions. By leveraging phage-host interactions and polymer binding properties, the polymer-phage sensor overcomes the limitations of traditional “lock-and-key” biosensors, offering enhanced specificity and reliability. This platform's rapid response time and adaptability make it a promising tool for clinical diagnostics and public health applications, particularly in combating antibiotic-resistant bacteria.

## Abstract 2

Pathogenic bacteria, such as methicillin-resistant *Staphylococcus aureus* (MRSA), pose significant challenges to public health due to their resistance to conventional antibiotics.

## Graphical abstract

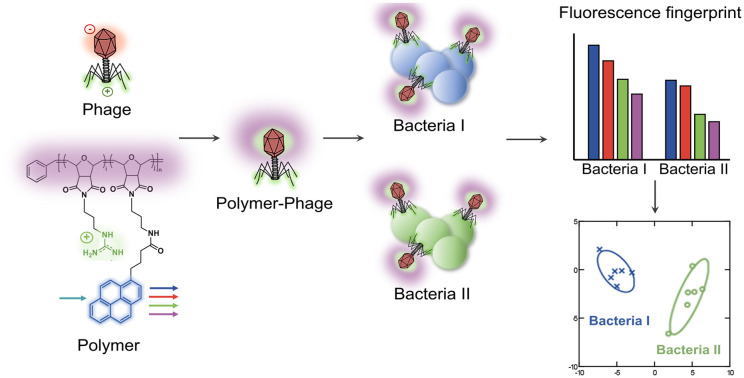

## Introduction

Pathogenic bacteria are widespread in our environment and pose a significant threat to public health. Among these, methicillin-resistant *Staphylococcus aureus* (MRSA) is identified by the World Health Organization (WHO) as a priority pathogen due to its resistance to conventional antibiotics and its capacity to cause severe infections.^1^ Early detection of both methicillin-sensitive *Staphylococcus aureus* (MSSA) and MRSA is crucial for the timely and effective administration of targeted treatments for the corresponding infections in clinical settings.^2^

Current diagnostic methods, such as culture-based assays and polymerase chain reactions, are often time-intensive, costly, and insufficiently generalized for reliable strain differentiation.^3,4^ Mass spectrometry-based omics approaches provide detailed protein, DNA, and metabolite profiles for bacterial fingerprinting but require complex and time-consuming pretreatments and complex data analysis.^5^ Surface-enhanced Raman spectroscopy (SERS) is another fingerprinting method for microbial identification. However, SERS depends on specialized active substrates and faces substrate homogeneity and reproducibility challenges. These limitations hinder effective infection control in high-stakes environments, such as hospitals, and prevent use in point-of-care situations.^6^ There is an urgent need for novel diagnostic tools that offer rapid, high-throughput, and precise bacterial identification.

Sensors are a crucial tool for guiding the treatment of bacterial infections, offering rapid and cost-effective identification of pathogens.^7^ Biosensors incorporating biological recognition elements can enhance detection capabilities through specific biological interactions with target bacteria.^8^ These biorecognition elements, such as antibodies, nucleic acids, and enzymes, can improve the accuracy and sensitivity of bacterial identification.^9^ Among these recognition elements, bacteriophages (phages) are particularly promising biorecognition elements for bacterial detection.^10,11^ Phages are ubiquitous in nature, self-amplifying, and resilient biological entities with a natural ability to target and bind to their host bacteria.^12,13^ Utilizing phages in bacterial sensors can provide high species/strain specificity and accuracy, making them excellent candidates for reliable bacterial detection.^14^ The specificity of phages is both a strength and a weakness for bacterial sensing. Phage recognition is generally limited to a single species or even a single strain of bacteria, restricting their broader applicability in diagnostics.^15,16^ The creation of phage-based biosensors capable of detecting multiple bacterial species/strains and discriminating between different strains of the same species is challenging, with only a few examples reported to date.^17,18^

Bacterial surfaces feature polysaccharides, proteins, and lipids. These biomolecules exhibit diverse chemical properties, such as varying surface charges and hydrophilic or hydrophobic groups. This diversity enables the use of materials with distinct characteristics to interact with bacteria, facilitating the development of biosensor arrays for bacterial detection.^19,20^ Noncovalent interactions, such as electrostatic and hydrophobic interactions, play a crucial role in bacterial recognition at the bacteria–material interface, serving as a foundation for designing sensor arrays.^21^ Array-based biosensors are effective and powerful tools for bacterial identification, leveraging versatile recognition mechanisms to detect subtle differences in bacterial samples.^21–23^ These biosensors operate by transforming chemical or physical properties of bacterial species into analytically useful detectable signals, often drawing inspiration from the mammalian olfactory system or the “chemical nose”.^24,25^ These signals are processed using statistical methods, generating distinct patterns that can discriminate various bacterial species, even identifying subtle differences in surface physicochemical properties or metabolites.^26^ Compared to traditional single-component biosensors, array-based platforms provide cross-reactive capabilities, enabling the simultaneous detection of multiple species and overcoming the specificity limitations of traditional phage-based sensors. The advantages of polymer-based sensors include scalability, stability, and structural tunability, which enable precise modulation of bacteria-polymer interactions in a customizable manner.^27–29^ Integrating phage recognition with array-based sensing offers the potential for synergistic integration of specific and selective recognition, making these hybrid sensor platforms promising for bacterial diagnostics.

Here, we report the development of a polymer-phage nanoassembly sensor array featuring rapid and sensitive bacterial detection. This multi-modal sensor array combines the specificity of phage-based recognition with the selective sensing capabilities of fluorescent polymers,^24^ enabling the discrimination of multiple bacterial species and strains. This sensor successfully differentiates three different species of bacteria – *Staphylococcus aureus*, *Escherichia coli*, and *Bacillus subtilis*, and three different clinical isolate strains of *S. aureus*, including one strain of MSSA and two strains of MRSA. Linear discriminant analysis (LDA) of the fluorescent signals obtained from the polymer-phage sensor provides enhanced discrimination between different bacterial species and strains compared to the polymer-only arrays. With rapid response times (minutes) and high-throughput potential, this biosensor platform is particularly well-suited for applications in public health and clinical diagnostics, where accurate differentiation between MSSA and MRSA is essential for effective infection control and patient safety.

## Results and discussion

### Synthesis and characterization of poly(oxanorbornene)-C3-Guan-pyrene polymer (PONI-C3-Guan-Py)

The sensing platform was engineered to provide an information-rich, four-channel output with only two sensor recognition elements. The first element of the sensor served both recognition and transduction roles: a cationic poly(oxanorbornene) (PONI) random copolymer scaffold functionalized with guanidine (Guan) groups and pyrene (Py) dye molecules. The polymer was synthesized by ring-opening metathesis polymerization using a third-generation Grubbs catalyst and was characterized by nuclear magnetic resonance (NMR) (*cf.* ESI†). Molecular weights and polydispersity index (PDI) were determined by tetrahydrofuran gel permeation chromatography (THF-GPC) (Fig. S1†). The positively charged guanidine groups facilitated selective interactions with negatively charged molecules, such as phage capsids or components (*e.g.*, lipids and polysaccharides) exposed at the bacterial surface.^30–32^ The solvatochromic pyrene molecules exhibit fluorescence from both their monomer and excimer states, which alter their spectral properties in response to changes in the local environment.^33^ The pyrene units provided four distinct fluorescence output signals at 378 nm, 398 nm, 420 nm, and 464 nm upon excitation at 346 nm (Fig. S2†).

### Self-assembly of a hybrid polymer-phage sensor

The second element of the sensor was a phage virion. We chose phage K due to its capacity to specifically capture and infect various strains of *S. aureus*, including MRSA.^34,35^

**Fig. 1:**
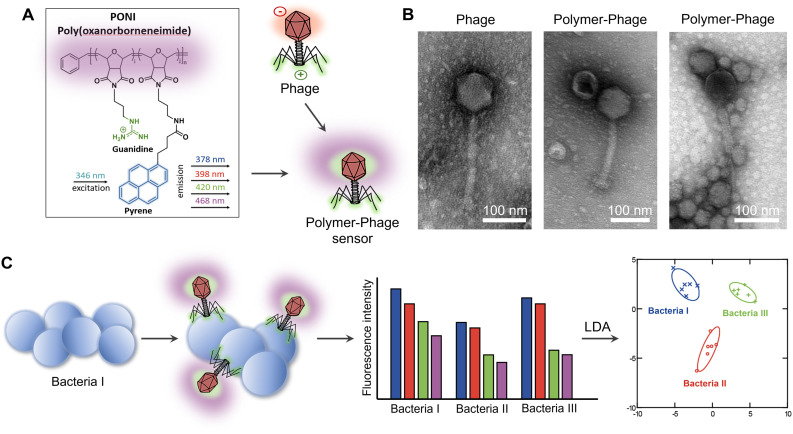
Design and working principle of the polymer-phage sensor. A). Chemical structure of poly(oxanorborneneimide) (PONI), functionalized with guanidine groups and a pyrene fluorophore. The schematic illustrates the interaction between the bacteriophage and the polymer, where the positively charged guanidine groups facilitate electrostatic interactions with the negatively charged phage capsid, creating the polymer-phage sensor. B). Transmission electron microscopy (TEM) images of individual phage K and polymer-phage assemblies. C). Schematic representation of the sensing mechanism. Interaction of the polymer–phage sensor with different bacterial species generates distinct fluorescence response patterns, enabling bacterial discrimination through multivariate statistical analysis.

We first tested the interaction of the PONI-C3-Guan-Py polymer (Fig. 1A) against phage K to establish retention of phage infectivity. Phages (∼10^9^ PFU mL^−1^) were mixed with the polymer to achieve final concentrations ranging from 0.25 μM to 4 μM (Table S1†). Afterward, the mixture was incubated for 30 minutes at 37 °C and titered using a modified double agar overlay assay. The results showed that the phages retained infectivity across all tested polymer concentrations (Fig. S3†), demonstrating polymer biocompatibility and suitability for integration into the sensor platform.

Dynamic light scattering (DLS) data confirmed the formation of the polymer-phage assembly, with size measurements of 38 nm for the polymer, 287 nm for the phage, and 656 nm for the polymer-phage complex (Fig. S4†). The increase in size is consistent with the electrostatic interactions and stable complex formation between the polymer and phage. Similarly, zeta potential measurements indicated changes in charge, shifting from +37 mV (polymer) and −12 mV (phage) to +5 mV for the polymer-phage assembly, further validating the stable sensor formation (Fig. S5†). Fluorescence spectra were recorded for the polymer-phage sensor with different phage concentrations (Fig. S6†). Transmission electron microscopy (TEM) provided direct visualization of these interactions (Fig. 1B). The left panel shows the morphology of phage K without the polymer added, featuring an icosahedral head (capsid) and a long contractile tail. The middle panel demonstrates polymers bound to the capsid. The right panel shows multiple polymer particles attached to the capsid, confirming that PONI-C3-Guan-Py was electrostatically interacting with the phage K capsid.

### Differentiation of three different bacterial species using the polymer-phage sensing platform

With the structural and functional properties of the polymer-phage assembly validated, we evaluated its performance in differentiating bacterial species and strains. Accurate and rapid differentiation of bacterial species is critical for clinical diagnostics and environmental monitoring.^36^ Identifying pathogens quickly can help guide targeted treatments and infection control strategies.^37^ To evaluate the performance of the polymer-phage sensing platform, we tested its ability to differentiate three bacterial species: *S. aureus*, *E. coli*, and *B. subtilis*.

We tested two MOIs (multiplicity of infection - the ratio of phages to bacteria) and two sensing time points to optimize sensor performance. The sensor was prepared by mixing 10 μL of polymer with 90 μL of phage solution, yielding final concentrations of 1 μM PONI-C3-Guan-Py and phage counts of 10^5^ PFU per well (MOI = 0.1) and 10^7^ PFU per well (MOI = 10). After a 30-minute incubation at room temperature to form the polymer-phage nanoassembly, 10 μL of dispersed (planktonic) bacteria (10^6^ CFU) were introduced to the sensor (Fig. 1C). The fluorescence emissions of the sensor, spanning four output channels, were measured hourly for 5 hours.

**Fig. 2:**
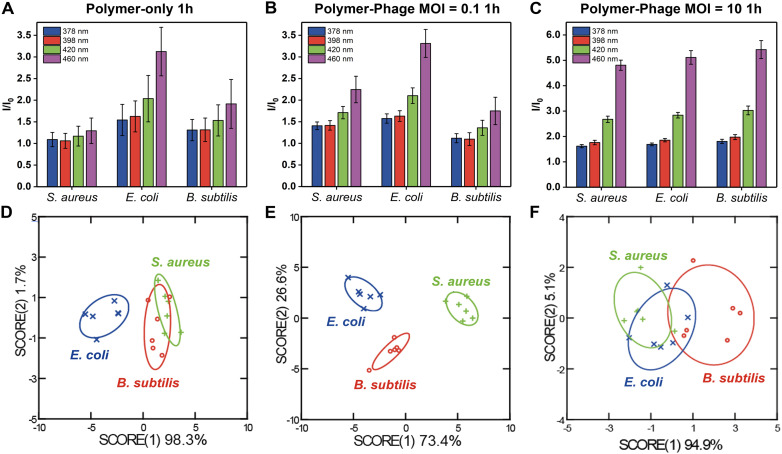
Differentiation of different bacterial species after 1-hour using polymer-only and polymer-phage sensors. A–C). Fluorescence signals of the four sensor channels normalized to the sensor only (*I*/*I*_0_). D–F). LDA plot of the first two canonical scores with 95% confidence ellipses. All of the experiments include six biological repetitions.

The sensor array exhibited distinguishable fluorescence signatures for different bacterial species, with notable differences in fluorescence intensity across the four measured wavelengths (378 nm, 398 nm, 420 nm, and 440 nm). The presence of phages at different multiplicities of infection (MOI) influenced the fluorescence response, demonstrating a substantial variation between the polymer-only sensor (Fig. 2A) and the polymer-phage sensor (Fig. 2B and C). The polymer-only sensor achieved a classification accuracy of only 61% (Fig. 2D, Tables S2 and S3†). The accuracy did not improve after 5 hours of incubation (61%). While the polymer-only sensor successfully distinguished *E. coli* from *S. aureus* and *B. subtilis*, the sensor could not differentiate between the two Gram-positive species. We hypothesized that the polymer-only sensor differentiated Gram-negative bacteria from Gram-positive bacteria depending on the differences in bacterial cell wall structure.^24^ This discrimination could be attributed to the interactions between the positively charged guanidine groups of PONI-C3-Guan-Py and the negatively charged components on bacterial surfaces.^24^ The cell walls of the Gram-negative and Gram-positive bacteria differ in their thickness, surface chemistry, lipid and lipoprotein contents, and receptors.^38,39^ Bacteria with different surface properties have different binding affinities with polymers.^40^ Functionalized polymers, such as PONI-C3-Guan-Py, undergo a conformational transformation due to the multivalent specific interaction with bacteria.^25^ Conformational changes of PONI-C3-Guan-Py in response to different bacterial surfaces lead to distinct fluorescence fingerprints.^24^ However, these differences were insufficient for accurate classification of two Gram-positive bacteria, *i.e.*, *S. aureus* and *B. subtilis*.

In contrast, the polymer-phage sensor provided the best differentiation of tested bacteria, achieving a classification accuracy of 100% at the MOI = 0.1 (Fig. 2E, Tables S4 and S5†). MOI = 10 resulted in a lower classification accuracy of 50% (Fig. 2F, Tables S6 and S7†). This decrease can be attributed to more pronounced bacterial lysis by the higher titer of phages, which disrupted the sensing process. The polymer-to-phage ratio at MOI = 0.1 likely facilitated stable interactions, enhancing sensor specificity. Compared to the polymer-only sensor, the clear differentiation between *S. aureus* and *B. subtilis* was attributed to phage K's host-specific binding capabilities, enhancing sensor specificity. Phages identify their appropriate host using receptor-binding proteins (RBPs), structures including tail fibers, head fibers, tail spikes, or a central tail spike.^41^ RBPs recognize specific molecules exposed on host bacteria surfaces, such as outer membrane proteins, lipopolysaccharides, teichoic acids, capsular polysaccharides, and organelles (*e.g.*, flagella or pili). Our results indicated that the critical role of phage–host interactions was retained in the complexes, improving bacterial differentiation.

### Differentiation of *S. aureus* strains using polymer-phage sensing platform

Building on the success of polymer-phage complexes in differentiating distinct bacterial species, we extended our investigation to test its ability to distinguish between closely related strains of *S. aureus*. Accurately distinguishing between strains of *S. aureus*, including methicillin-resistant (MRSA) and methicillin-sensitive variants (MSSA), is crucial for guiding appropriate antibiotic treatments and managing infection control.

We tested two sensing time points at MOI of 0.1 to optimize sensor performance. The sensor was created by adding 10 μL of polymer to 90 μL of phage solutions, resulting in final concentrations of 1 μM PONI-C3-Guan-Py and 10^6^ PFU per well. After a 30-minute incubation at room temperature to form the polymer-phage nanoassembly, 10 μL of bacteria (10^7^ CFU) were introduced to the sensor. Fluorescence emissions across four output channels were measured at 30 and 60 min. Unknown sample identification was performed to validate the sensor's reliability by comparing results against the training set.

**Fig. 3:**
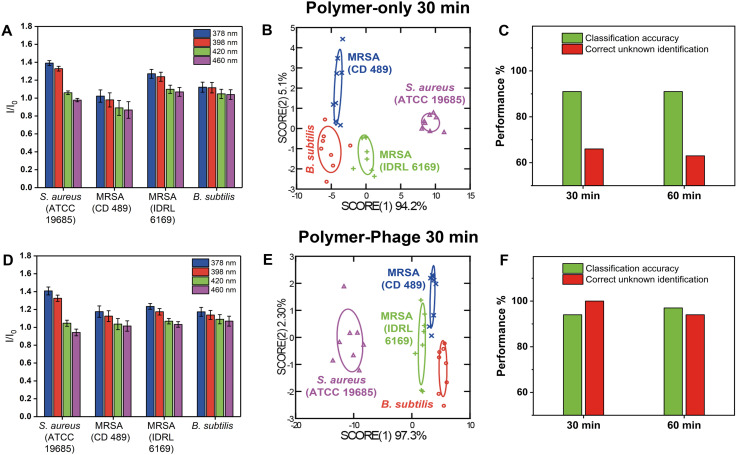
Comparison of polymer-only and polymer-phage sensors in differentiating Gram positive bacteria, including different strains of the same species, after 30 minutes. A and D). Fluorescence signals of the four sensor channels were normalized to the sensor only. Fluorescence intensities of each treatment group were obtained at 30 min and normalized against the sensor only. All the experiments included eight biological repetitions. B and E). LDA of the first two canonical score plots of the fluorescence response patterns. All the experiments included eight biological repetitions. C and F). Classification accuracy and correct unknown identification of *B. subtilis* and three different strains of *S. aureus* after 30 min and 60 min.

The polymer-only sensor generated distinct fluorescence patterns for each bacterial species (Fig. 3A), achieving a classification accuracy of 91% within 30 minutes (Fig. 3B, Tables S8 and S9†). In these experiments, the bacterial load was 10^7^ CFU per well, an order of magnitude higher than in the species-level experiments (*cf.* section ‘Differentiation of three different bacterial species using the polymer-phage sensing platform’), which enhanced polymer–bacteria interactions and yielded more pronounced fluorescence responses. However, despite its ability to generate distinct fluorescent patterns at elevated concentrations, the polymer-only sensor demonstrated low reliability in unknown sample identification, with a correct unknown identification (CUI) rate of only 66% at 30 min and 63% at 60 min (Fig. 3C, Tables S10 and S14–S16†). This suggested that while PONI-C3-Guan-Py polymer interacted with bacterial surfaces, its non-specific binding was still limiting consistent differentiation of closely related strains such as MRSA and MSSA, even at higher bacterial counts.

The polymer-phage sensors exhibited distinguishable fluorescence signatures across different bacterial species (Fig. 3D). In contrast to the polymer-only sensor, the polymer-phage sensor exhibited excellent differentiation results between Gram-positive bacteria (Fig. 3E), among which three were hosts for the used phage. We achieved a classification accuracy of 94% and a CUI rate of 100% within 30 minutes (Fig. 3F, Tables S11–S13†). These results were attributed to the specificity provided by phage–host interactions. Phage K's receptor-binding proteins (RBPs) specifically targeted and bound to *S. aureus* strains, triggering conformational changes in the phage that enhanced the stability of the interaction.^42^ This specificity complemented the PONI-C3-Guan-Py polymer's broad surface interactions, resulting in a dual-mode sensor that captured both general bacterial surface features and strain-specific molecular interactions. At MOI = 0.1, the polymer-to-phage ratio was optimal for achieving stable interactions without rapid bacterial lysis, which would otherwise disrupt the sensing process. Even after 60 minutes, the polymer-phage sensor maintained a high classification accuracy of 97% and a CUI rate of 94%, demonstrating its robustness and reliability over time (Fig. 3F, Tables S17–S19†).

In Gram-positive bacteria such as *S. aureus*, wall teichoic acids (WTAs) are prominent components of the cell wall. These anionic polymers, composed of phosphate-linked polyalcohol chains anchored to the peptidoglycan layer, serve as key receptors for many staphylococcal phages.^43^ Phage K adsorbs to its bacterial host through the receptor-binding protein Gp144, which is located in the baseplate region of the phage tail. Gp144 specifically binds to the phosphate-rich backbone of WTAs, rather than to their glycosylated side chains.^44^ This mode of recognition is governed by the structural complementarity between Gp144 and the conserved WTA backbone, allowing phage K to selectively identify *S. aureus* cells.^45^ The specificity of Gp144 was demonstrated in a study by Semra *et al.*, in which recombinant Gp144 was fluorescently labelled and incubated with various bacterial strains. The labelled protein bound exclusively to *S. aureus* and MRSA cells, showing no detectable interaction with *E. coli*, *Enterococcus faecalis*, or *Bacillus cereus*. Their results confirm that Gp144-mediated recognition is both genus- and species-specific, and support its role in the strain-level discrimination observed in our polymer-phage sensor.

The limit of detection (LOD) was established by conducting experiments at different concentrations of *S. aureus* (ranging from 10^2^ to 10^5^ CFU) against varying phage concentrations (10^3^ to 10^6^ PFU). Fluorescence signals were measured after 1 hour of incubation and analysed by LDA. Phage concentrations below 10^3^ PFU per well might not generate distinct fluorescence signals, whereas concentrations above 10^7^ PFU per well could rapidly lyse the host bacteria, given the tested bacterial range of 10^2^ to 10^5^ CFU per well. We determined that the LOD of our polymer-phage biosensor is approximately 100 CFU per well without the bacteria amplification process. The optimal conditions for bacterial differentiation were determined to be 1 μM polymer combined with 10^3^ PFU, yielding the highest classification accuracy of 75% (Fig. S7†).

The ability of the polymer-phage sensor to differentiate between *S. aureus*, MRSA CD 489, MRSA IDRL 6169, and *B. subtilis* highlighted the critical role of phage-host interactions in enhancing bacterial differentiation. While the polymer-only sensor relied solely on electrostatic interactions and conformational changes, incorporating phages introduced a layer of molecular specificity that significantly improved accuracy and reliability. Compared to existing phage-based biosensors reported in the literature (Table S20†), our polymer-phage sensor uniquely differentiates multiple bacterial species as well as closely related strains simultaneously without amplification, providing rapid results within approximately 1–1.5 hours. These findings underscored the potential of the polymer-phage sensing platform as a powerful diagnostic tool for differentiating bacterial strains, particularly in addressing the growing challenge of antibiotic resistance.

## Conclusions

This study presents a polymer-phage sensor platform capable of rapid and accurate bacterial detection and differentiation. The sensor demonstrates outstanding performance, achieving high classification accuracy (94–100%) and correct unknown identification rates (94–100%) across multiple bacterial species, including *S. aureus*, *E. coli*, and *B. subtilis*, as well as between closely related *S. aureus* strains (MSSA and MRSA). The dual functionality of the sensor combines the host specificity of phage with the versatile selective recognition capabilities of a functionalized polymer. This integrated recognition process enables the detection of subtle differences in bacterial surface structures and composition. The results highlight the sensor's ability to overcome the limitations of traditional “lock-and-key” biosensors, which often lack sensitivity and adaptability for complex bacterial mixtures. The polymer-phage sensor provides a robust and reliable diagnostic tool, offering fast response times and reproducible results, particularly in addressing the growing challenge of antibiotic-resistant pathogens. Future work will focus on validating the sensor's performance in complex biological samples to advance its integration into clinical diagnostic systems and infection control strategies.

## Experimental

### Materials

Chemicals and solvents for syntheses were purchased from Fisher Scientific and Millipore Sigma and used as received unless stated otherwise. Absorbance and fluorescence measurements were carried out using a SpectraMax M2 plate reader (Molecular Devices, San Jose, CA, USA).

### PONI-C3-guanidine-pyrene synthesis and characterization

The polymer was synthesized according to previous reports.^32^ Details are provided in the ESI.†

### Phage propagation and quantification

Bacterial colonies were grown in tryptic soy broth (TSB) and incubated at 37 °C with shaking at 220 rpm for 2–3 hours until reaching the mid-log phase (OD_600_ = 0.4–0.6, corresponding to approximately 10^7^ CFU mL^−1^). The mid-log phase bacterial culture was then transferred into a fresh tube containing cation-supplemented TSB (10 mM MgSO_4_) and mixed with a phage K stock. The mixture was gently agitated and incubated at 37 °C with mild shaking (120 rpm) for 4 hours or until the culture cleared. Following incubation, the cultures were centrifuged to remove bacterial cells, and the supernatant was passed through a 0.22 μm filter. The filtered supernatant was then titered using a modified double agar overlay assay. Filtrates were stored at 4 °C and re-titered before further use.

A 150 μL aliquot of bacterial culture was mixed with cooled molten overlay agar, ensuring the temperature remained above 55 °C to prevent premature solidification. The mixture was gently tapped to ensure even distribution and then poured over the underlay agar layer of a Petri dish. Next, 10 μL aliquots of phage diluent were spotted on the surface and allowed to solidify at room temperature for 10 minutes. The plates were incubated overnight at 37 °C, and plaques were counted.

### Bacteria

Frozen bacterial species and strains (−80 °C), including *E. coli* (CD-2), *B. subtilis* (FD6b), *S. aureus* (ATCC 19685), MRSA (IDRL-6169), and MRSA (CD-489), were aerobically cultured on Luria-Bertani agar. Overnight cultures were prepared by transferring a single colony from the agar plate into sterile LB broth in culture tubes. These cultures were incubated at 37 °C with agitation (275 rpm) until reaching the stationary phase. Bacteria were then harvested by centrifugation (7000 rpm, 5 minutes) and washed thrice with 0.85% sodium chloride. The bacterial pellets were resuspended in 1 mL of PBS, and the OD600 was measured using a SpectraMax M2 (Molecular Devices). Clinical isolates labeled with “CD” were sourced from Cooley Dickinson Hospital (Northampton, MA, USA), and “IDRL” originated from the Infectious Diseases Research Laboratory at Mayo Clinic (Rochester, MN, USA).

### Transmission electron microscopy (TEM)

Phage morphology and polymer-phage assemblies were visualized by transmission electron microscopy. Briefly, 5 μL of phage or polymer-phage suspension was deposited onto 400-mesh copper grids (Sigma-Aldrich) pre-coated with 2% collodion solution and a carbon layer. After allowing phages to adsorb, grids were washed, stained with 2% uranyl acetate (pH 4.5; BDH Chemicals, UK), and air-dried. Imaging was performed at 120 kV on an FEI Tecnai G2 Spirit BioTWIN TEM, and micrographs were recorded using Olympus Soft Imaging Solution software.

### Array-based sensing procedures

The polymer-phage sensor was prepared by adding 10 μL of PONI-C3-Guan-Py polymer solution to 90 μL of phage solutions with varying concentrations (resulting in 10^5^ to 10^7^ PFU per well) to achieve MOI values of 0.1 and 10 after the addition of bacteria. The polymer-phage mix was incubated at room temperature for 30 minutes to form the polymer-phage nanoassembly. To test the sensor's performance, 10 μL of bacteria (*S. aureus* ATCC 19685, MRSA CD 489, MRSA IDRL 6169, *E. coli*, and *B. subtilis*, resulting in 10^6^ to 10^7^ CFU per well) were added to the sensor in microwells. Fluorescence emissions were recorded across four output channels (346/378 nm, 346/398 nm, 346/420 nm, and 346/464 nm) at 30 minutes, 1 hour, and 5 hours, depending on the experiment. Unknown sample identification was performed to verify the sensor's reliability by comparing results to the training set for experiments differentiating *S. aureus* strains. DLS and zeta potential of polymer, phage, and polymer-phage assembly were measured by the Zetasizer Nano ZS instrument (Malvern).

### Linear discriminant analysis (LDA)

Discrimination analysis was performed using SYSTAT software (version 12.0). For bacteria sensing in the microplate, the raw data contained a matrix of 6 (replicates) × 3 (bacteria) × 4 (channels) in the case of *S. aureus*, *E. coli*, *B. subtilis*, and 8 (replicates) × 4 (bacteria) × 4 (channels) in case of *S. aureus* ATCC 19685, MRSA CD 489, MRSA IDRL 6169, *B. subtilis*.

Normalized fluorescence data were analyzed using LDA to classify groups and statistically differentiate fluorescence responses across bacterial targets. The analysis was conducted in complete mode with a tolerance set at 0.001, utilizing all variables. LDA enhanced the ratio of between-class variance to within-class variance, allowing for optimal group separation. Input data were transformed into canonical scores or factors, which were linear combinations of the response patterns, to best separate the groups.

This transformation reduced the dimensionality of the data, and the first two canonical factors, which accounted for the largest percentages of variance, were illustrated in Fig. 2 and 3. A 2D plot visually represented the positioning of each data point in the transformed dimensional space, where the axis values are mathematical constructs with no physiological significance. This method differentiated bacterial fluorescence responses within the multichannel system.

### Unknown identification

The identity of unknown samples was determined by calculating the Mahalanobis distance to the training groups using LDA. Each unknown sample was represented by the average response from eight data points. During LDA, the normalized fluorescence responses of the unknown samples were first converted into canonical scores using the discriminant functions established from the reference set. Then, the Mahalanobis distance of the unknown sample to the centroid of each group generated by the training set was computed in the LDA space. The unknown sample was classified into groups with the shortest Mahalanobis distance, indicating the closest match.

## Conflicts of interest

There are no conflicts to declare.

## Supplementary Material

SD-004-D5SD00069F-s001

## Data Availability

Data for this article are available at RepOD at https://doi.org/10.18150/7IVYGW. The data supporting this article have also been included as part of the ESI.†
