# Supplementary material for: Array-based polymer-phage biosensors for detection and differentiation of bacteria
Source: Sens Diagn. 2025 Jul 2;4(9):759–66. doi: 10.1039/d5sd00069f (PMC12235245; doi:10.1039/d5sd00069f)
Supplement: SD-004-D5SD00069F-s001 [file SD-004-D5SD00069F-s001.pdf]

Supplementary Information (SI) for Sensors & Diagnostics.
This journal is © The Royal Society of Chemistry 2025

SUPPORTING INFORMATION

Array-based Polymer-Phage Biosensor for Detection and Differentiation of Bacteria

Enkhlin Ochirbat,[a] Junwhee Yang,[b] Aritra Nath Chattopadhyay,[b] Jungmi Park,[b] Mingdi Jiang,[b] Jan Paczesny,*[a] Vincent M. Rotello*[b]

[a] *Institute of Physical Chemistry, Polish Academy of Sciences, Kasprzaka 44/52, 01224 Warsaw, Poland*

[b] *Department of Chemistry, University of Massachusetts Amherst, 710 N. Pleasant St., Amherst, MA 00103, USA*

[*] *Author to whom correspondence should be addressed jpaczesny@ichf.edu.pl, rotello@umass.edu*

Table of contents

1. Polymer & monomer synthesis
2. Characterization and compatibility of PONI-C3-Guan-Pyrene polymer and polymer-phage assembly
3. Sensing data
   3.1. Discrimination of three different bacteria species (*S. aureus*, *E. coli*, and *B. subtilis*)
   3.2. Discrimination and identification of *B. subtilis* and three different strains of *S. aureus* (*S. aureus* ATCC 19685, MRSA CD 489, MRSA IDRL 6169)
   3.3. Discrimination of bacterial concentrations
   3.4. Overview of phage-based biosensors and commercial diagnostic platforms

1. Polymer & monomer synthesis

Synthetic protocols for Compound **5**, Compound **6**, and **PONI-C3-Guan-Py** were slightly modified from previous reports.[1]

Synthesis of **1**

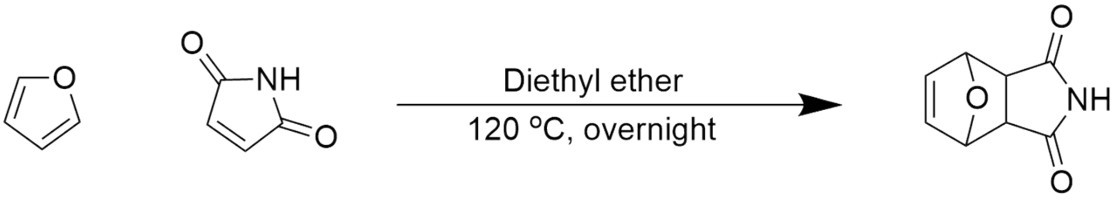

1

Maleimide (4.00 g, 41.1 mmol) and furan (4.20 g, 61.7 mmol) were added to a 250 mL pressure flask. Next, diethyl ether was added to the pressure flask. The flask was tightly sealed with Teflon tape, and the reaction mixture was heated at 120 ˚C overnight. Afterward, the reaction mixture was cooled to room temperature. Subsequently, the precipitate was filtered and rinsed with 100 mL of diethyl ether to remove residual furan and stored under vacuum to yield Compound **1**. Chemical yield: 84 %, white crystal. 1H NMR (400 MHz, DMSO-d6) δ 11.16 (s, 1H), 6.54 (tr, 2H), 5.12(tr, 2H), 2.85 (s, 2H).

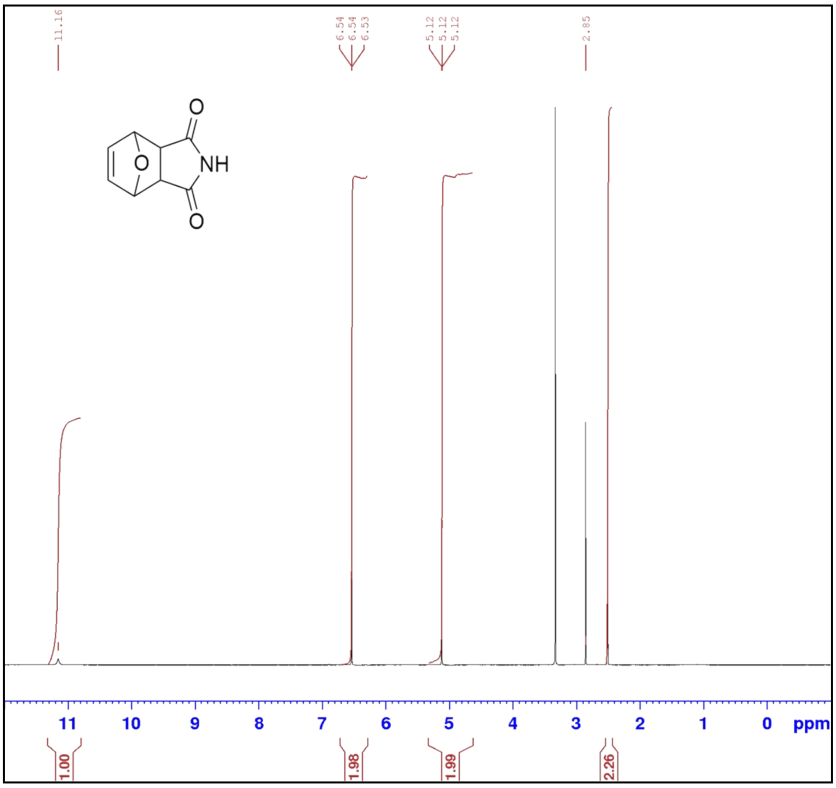

Synthesis of **2**

1 2

To a 500 mL round-bottom flask, compound **1** (1.00 g, 6.05 mmol), tert-butyl bromopropyl carbamate (1.72 g, 7.27 mmol), and 150 mL of acetonitrile were added. Subsequently, potassium carbonate (3.35 g, 24.2 mmol) and potassium iodide (0.02 g, 0.12 mmol) were added to the reaction mixture while it was vigorously stirred, and the mixture was heated to 70°C overnight. Afterward, the reaction mixture was cooled to room temperature, and the precipitate was vacuum-filtered. The filtrate was rotovaped, dissolved in 100 mL of ethyl acetate, and washed with brine (3 × 100 mL). The ethyl acetate layer was dried over magnesium sulfate and concentrated using a rotary evaporator. The crude product was then purified via silica gel chromatography (ethyl acetate/hexane 1:1) to obtain Compound **2**. Chemical yield: 73%, white solid. 1H NMR (400 MHz, CDCl3) δ 6.53 (s, 2H), 5.28 (s, 2H), 3.65, (t, 2H), 3.46 (t, 2H), 2.85 (s, 2H), 1.58 (m, 4H), 1.28 (m, 14H).

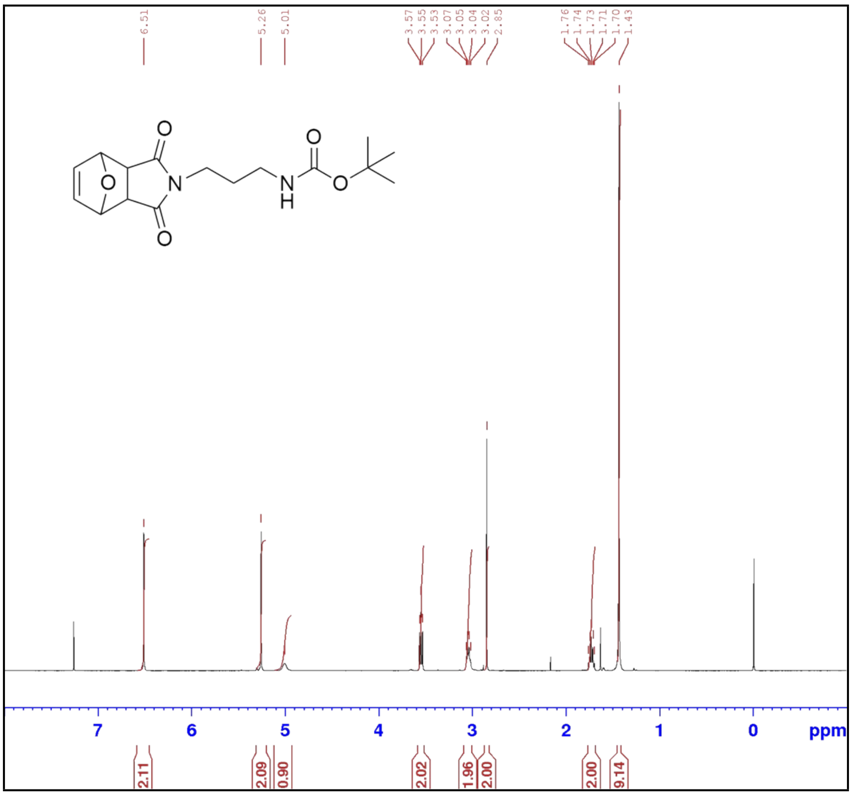

Synthesis of **3**

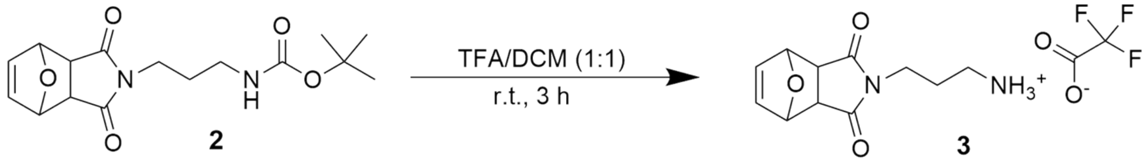

To a 50 mL round-bottom flask, Compound **2** (2.00 g, 3.10 mmol) was added and dissolved in 10 mL of dichloromethane. Trifluoroacetic acid (10 mL) was then slowly added to the reaction mixture, and the solution was stirred for 3 hours. Following the reaction, the crude mixture was concentrated under reduced pressure using a rotary evaporator. To the resulting residue, 30 mL of diethyl ether was added to precipitate the crude product. The diethyl ether layer was then removed by vacuum filtration to yield Compound **3**. Chemical yield: 95%, white solid. 1H NMR (400 MHz, DMSO-d6) δ 7.70 (s, 3H), 6.56 (s, 2H) 5.13 (s,2H), 3.43, (t, 2H), 2.94 (s, 2H), 2.74 (q, 2H), 1.73 (q, 2H).

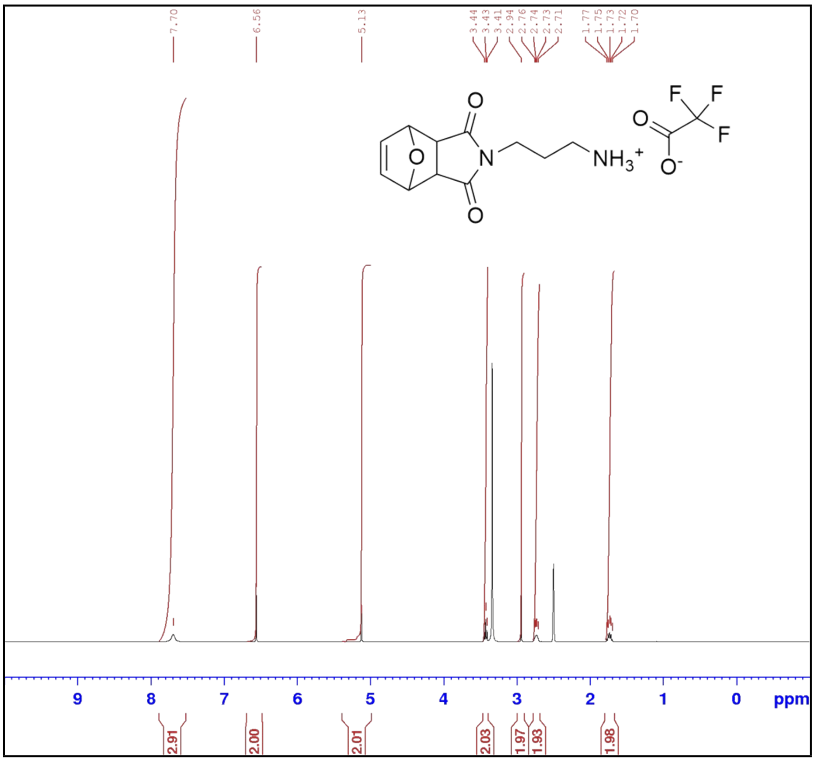

Synthesis of **4**

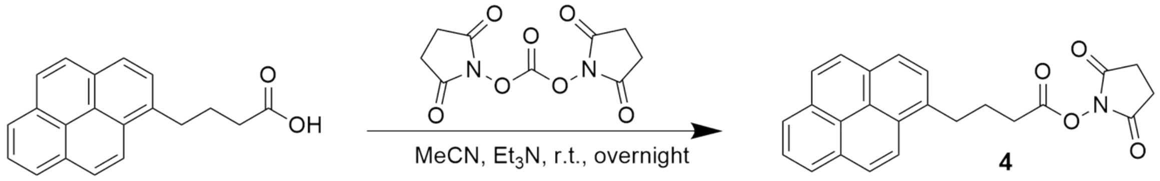

To a 250 mL round-bottom flask, 1-pyrenebutyric acid (0.50 g, 1.74 mmol), disuccinimidyl carbonate (1.11 g, 4.34 mmol), and 100 mL of acetonitrile were added. The reaction mixture was stirred, and triethylamine (967 µL, 6.96 mmol) was slowly added. The mixture was stirred overnight. After the reaction, the solvent was removed under reduced pressure using a rotary evaporator. The residue was dissolved in 100 mL of ethyl acetate and stored in a refrigerator for 1 day to precipitate excess disuccinimidyl carbonate. The precipitate was removed by vacuum filtration, and the filtrate was washed with brine (3 × 50 mL), dried over anhydrous magnesium sulfate, and concentrated under reduced pressure. The resulting product was used directly in the subsequent reaction without further purification.

Synthesis of **5**

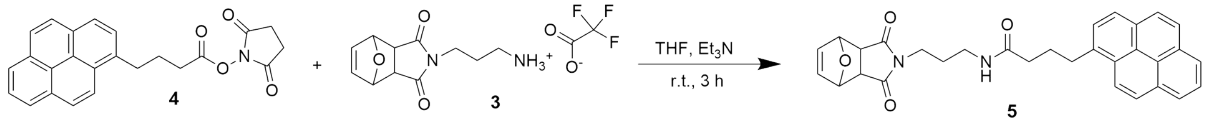

To a 100 mL round-bottom flask, Compound **3** (0.17 g, 0.52 mmol), Compound **4** (0.20 g, 0.52 REFmmol), and tetrahydrofuran were added. While stirring the reaction mixture, triethylamine (72.3 µL, 0.70 mmol) was slowly added. The reaction mixture was stirred for 3 hours. Afterwards, the reaction mixture was concentrated and dissolved in 50 mL of ethyl acetate. The organic layer was washed with brine (3 × 50 mL) and dried over magnesium sulfate. The organic layer was then concentrated, and the resulting crude product was further purified by silica gel chromatography (ethyl acetate/hexane, 2:1) to yield compound **5**. Chemical yield 59%, pale brown solid. 1H NMR (400 MHz, CDCl3) δ 8.30 (d, 2H), 8.15 (m,2H), 8.11, (s, 1H), 8.10 (d, 1H), 7.99 (m, 3H), 7.87 (d, 1H), 6.43 (s, 2H), 6.09 (t, 1H), 5.20 (s, 2H), 3.49 (s, 2H), 3.39 (t, 2H), 3.17 (q, 2H), 2.70 (s, 2H), 2.32 (m, 2H), 2.21(m 2H).

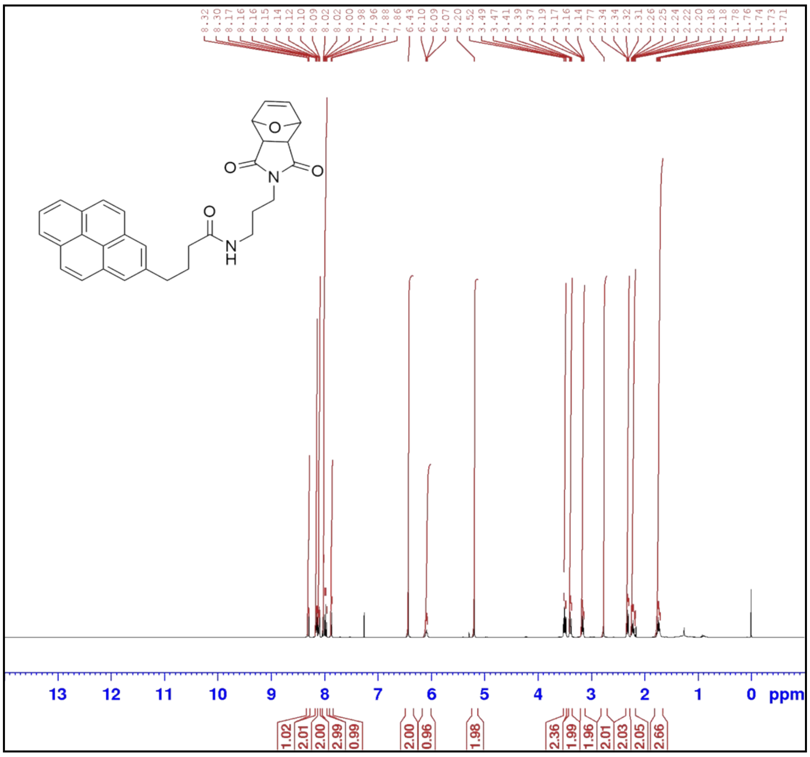

Synthesis of **6**

To a 250 mL round-bottom flask, Compound **3** (1.50 g, 4.48 mmol) and N,N′-di-Boc-1H-pyrazole-1-carboxamidine (2.09 g, 6.72 mmol) were added, followed by the addition of a 9:1 mixture of acetonitrile and water (50 mL). While stirring the reaction mixture, triethylamine (5.60 mL, 40.3 mmol) was slowly added and stirred overnight. The reaction mixture was then concentrated using a rotary evaporator and dissolved in 100 mL of ethyl acetate. Subsequently, the organic layer was washed with brine (3 x 100 mL) and dried over magnesium sulfate. The organic layer was rotovaped, and the resulting crude product was further purified by silica gel chromatography (ethyl acetate/hexane 1:1) to yield Compound **6**. Chemical yield 74%, white crystal. 1H NMR (400 MHz, CDCl3) δ 11.53 (s,1H), 8.49 (t,1H), 6.51, (s, 2H), 5.28 (s, 2H), 3.56 (t, 2H), 3.39 (q, 2H), 1.49 (d, 18H).

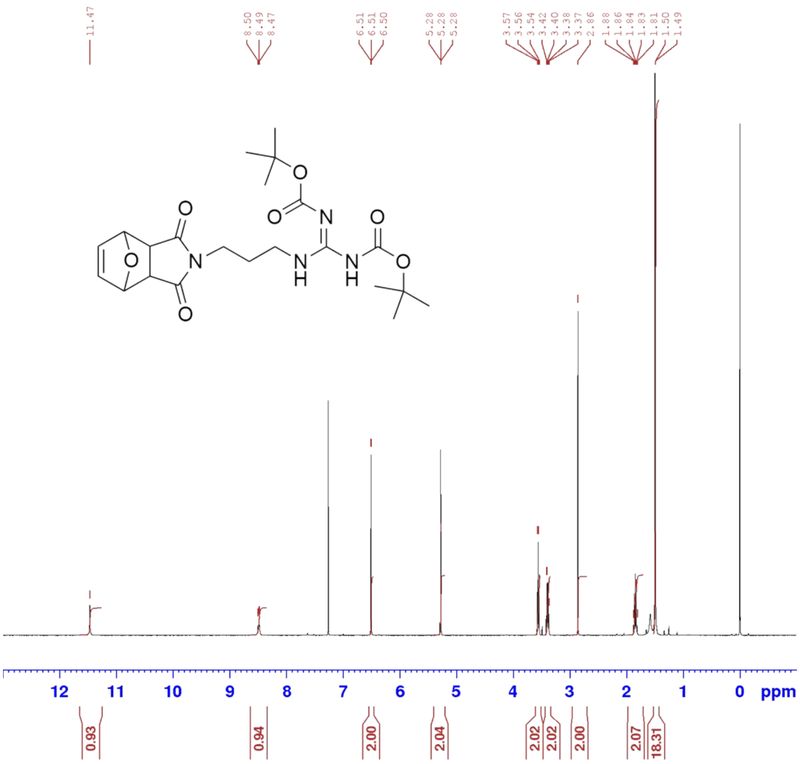

Synthesis of **PONI-C3-Guanboc-Py**

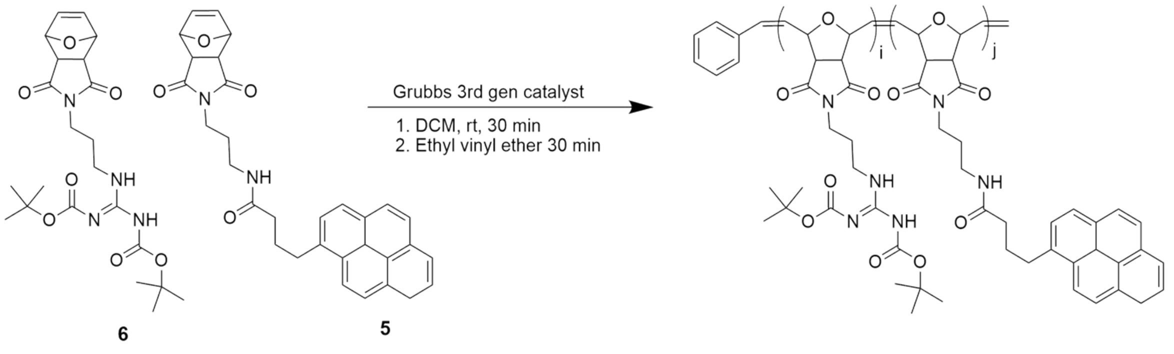

To a 10 mL Schlenk flask, a solution of Compound **5** (21.2 mg, 4.30 µmol) and **6** (200 mg, 0.43 mmol) in 3 mL of dichloromethane was added. In a separate 10 mL Schlenk flask, Grubbs 3rd generation catalyst was dissolved in 1 mL of dichloromethane. Both flasks were sealed with septa and degassed via freeze-pump-thaw (3 cycles). Subsequently, the Grubbs 3rd generation catalyst (4.19 mg, 4.73 µmol) solution was transferred to the Schlenk flask containing Compound **5** and Compound **6** to initiate the polymerization. The reaction mixture was then stirred for 1 hour under nitrogen. Next, ethyl vinyl ether (200 µL) was added, and the mixture was stirred for an additional 20 minutes to quench the polymerization. Afterwards, the polymer solution was passed through a short pad of aluminum oxide beads to remove the Grubbs catalyst. The resulting polymer solution was then concentrated and precipitated into a stirred diethyl ether/hexane (1:1). The precipitated polymer was filtered and dried in reduced pressure to yield **PONI-C3-Guanboc-Py**. 1H NMR (400 MHz, CDCl3) δ 11.48 (s,1H), 8.46 (br,1H), 7.88-8.34 (m, 0.65H) 6.10, (br, 1H), 5.78 (br, 1.2H), 5.05 (br, 1.2H), 3.57 (m, 2H), 3.42 (m, 2H), 3.08 (br, 0.14H), 1.88 (m, 2H), 1.50 (S, 18H)

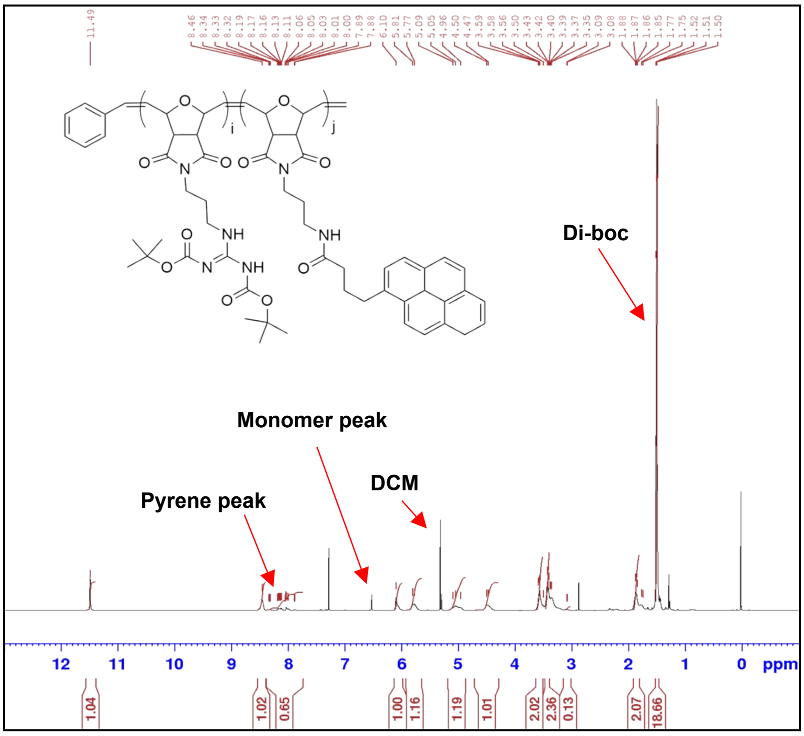

Synthesis of **PONI-C3-Guan-Py**

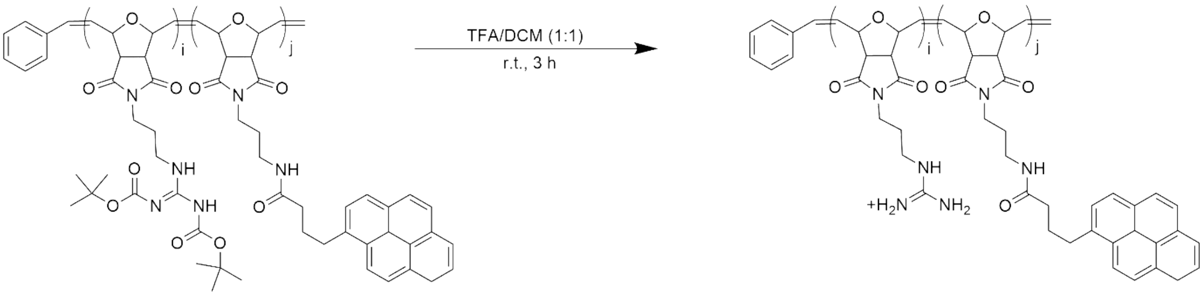

PONI-C3-Guanboc-Py **PONI-C3-Guan-Py**

To a 20 mL glass vial, 100 mg of the protected polymer was added and dissolved in 5 mL of dichloromethane. While stirring the polymer solution, 5 mL of trifluoroacetic acid was slowly added. The deprotection reaction proceeded for 3 hours. The reaction mixture was then concentrated in vacuo, and the resulting crude product was rinsed with diethyl ether (3 × 20 mL) to remove residual trifluoroacetic acid. The deprotected polymer was then dissolved in a minimal amount of deionized water and filtered through a 0.22 µm PES filter to remove any precipitate. The polymer solution was subsequently dialyzed against deionized water using a cellulose membrane (cut-off 10,000 Da). The dialysis water was changed five times at 4-hour intervals. Finally, the purified polymer was lyophilized to yield **PONI-C3-Guan-Py**.

2. Characterization and compatibility of PONI-C3-Guan-Pyrene polymer and polymer-phage assembly.

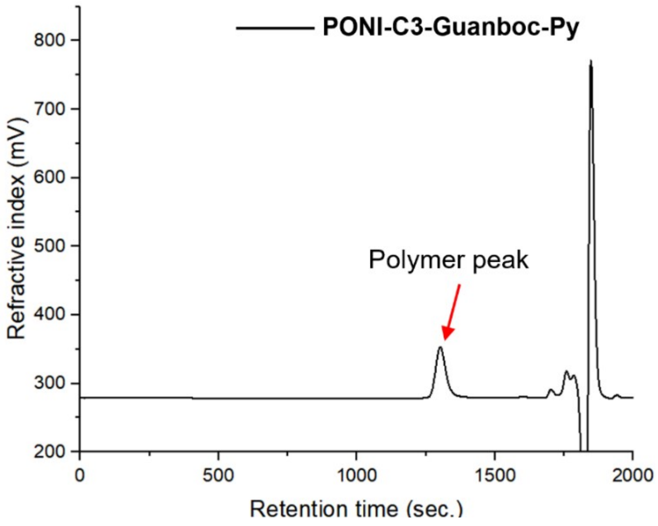

Figure S1. Characterization of Boc-protected polymer *via* gel permeation chromatography. GPC trace shows that **PONI-C3-Guanboc-Py** has Mw = 42.195 kDa, Mn = 39.883 kDa, and a polydispersity index of 1.055, determined by GPC using polystyrene standards, THF as the eluent, and toluene as the flow marker.

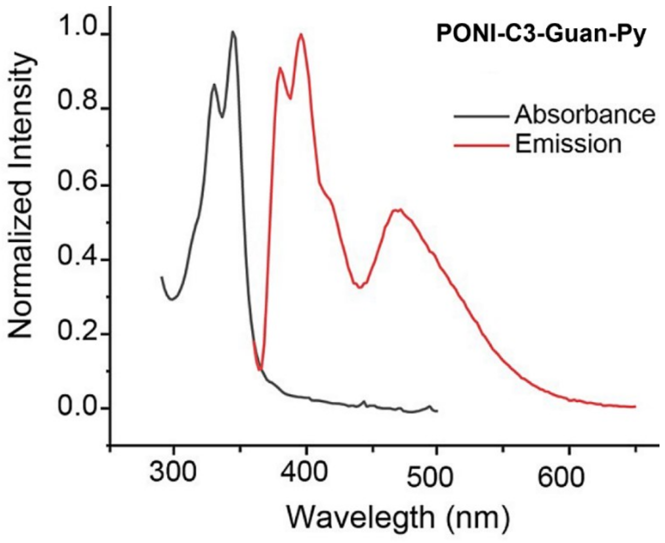

Figure S2. Optical properties of PONI-C3-Guan-Pyrene. Absorbance and emission spectra of PONI-C3-Guan-Py polymer were measured using Molecular Devices Spectramax M2 plate reader.

Table S1. Phage samples with varying final concentrations of PONI-C3-Guan-Pyrene.

PONI-C3-Guan-Pyrene (final concentration, uM)	***control***	***0.25***	***0.5***	***1.0***	***2.0***	***3.0***	***4.0***
PONI-C3-Guan-Py (stock, uM)	0	10	10	10	100	100	100
Phage K (stock, PFU/mL)	10^9	10^9	10^9	10^9	10^9	10^9	10^9
Phage K (uL)	90	90	90	90	90	90	90
PONI-C3-Guan-Py from stock (uL)	0	2.5	5.0	10.0	2.0	3.0	4.0
Buffer (uL)	10	7.5	5	0	8	7	6

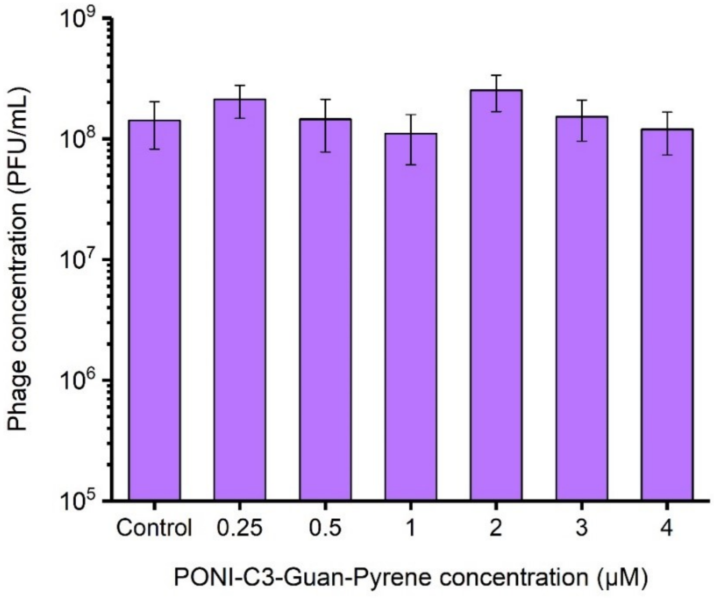

Figure S3. **Effects of PONI-C3-Guan-Pyrene polymer on phage viability**. PONI-C3-Guan-Py does not decrease the titer of phage K. All data represent eight technical replicates.

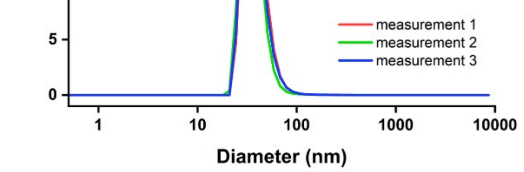

B

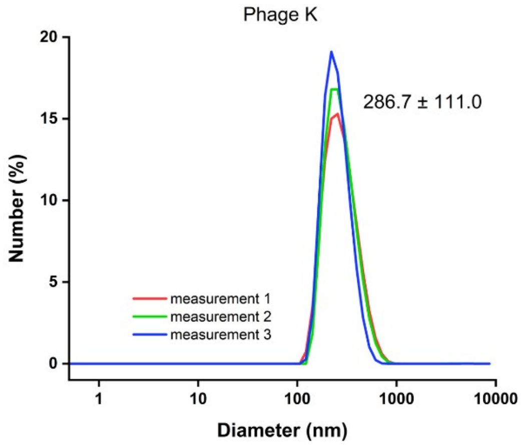

C

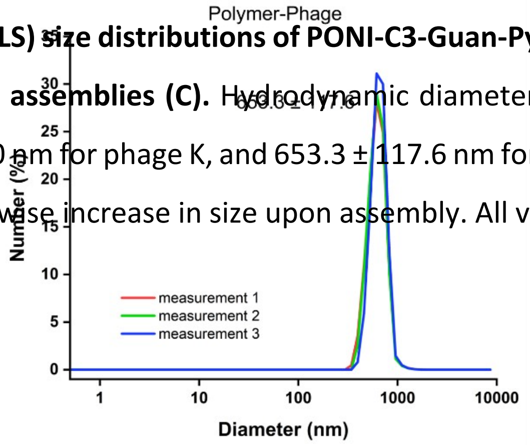

Figure S4. Dynamic light scattering (DLS) size distributions of PONI-C3-Guan-Pyrene polymer (A), phage K (B), and polymer-phage assemblies (C). Hydrodynamic diameters were 37.7 ± 12.6 nm for the polymer, 286.7 ± 111.0 nm for phage K, and 653.3 ± 117.6 nm for the polymer–phage complex, demonstrating a stepwise increase in size upon assembly. All values are from three independent measurements.

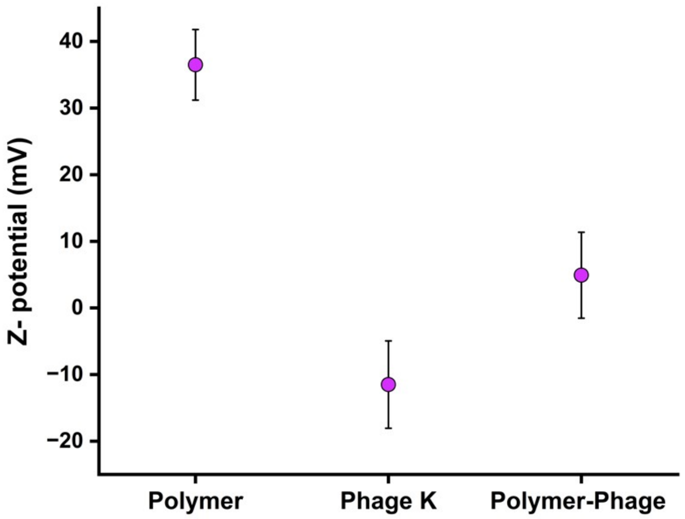

Figure S5. Zeta potential analysis of PONI-C3-Guan-Pyrene polymer, phage K, and polymer-phage assemblies. PONI-C3-Guan-Pyrene polymer exhibited +36.5 ± 5.3 mV, phage K -11.5 ± 6.56 mV, and polymer-phage assembly +4.9 ± 6.4 mV, consistent with the electrostatic binding. All data represent three independent measurements.

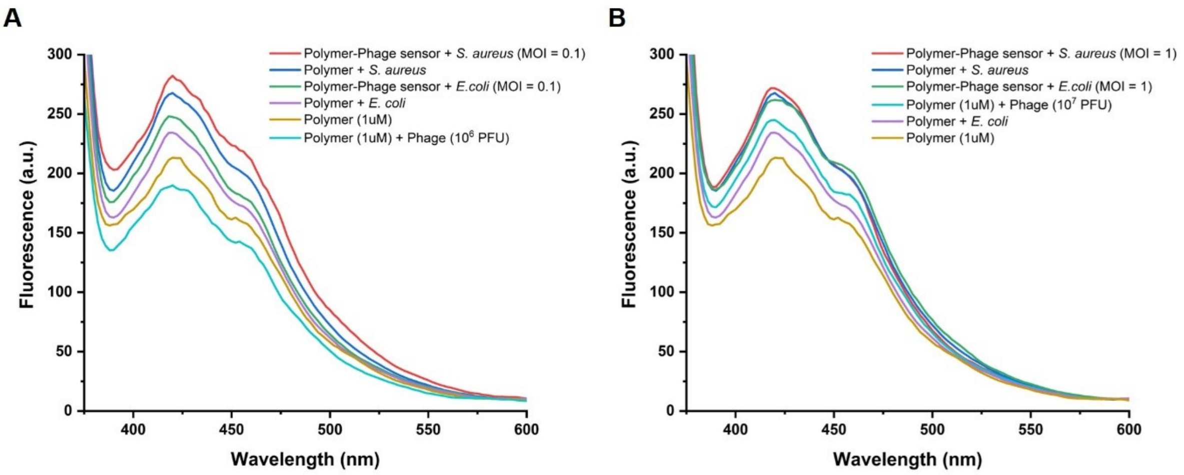

Figure S6. Fluorescence spectra of the polymer-phage sensor upon exposure to bacterial species in TSB medium. A) Spectra for sensors prepared with 1 µM polymer and a multiplicity of infection (MOI) of 0.1. B) Spectra for sensors with increased MOI of 1, showing comparatively smaller intensity differences between bacterial conditions. Data represent the mean of eight measurements.

3. Sensing data

3.1. Discrimination of three different bacterial species (*S. aureus*, *E. coli*, and *B. subtilis*)

Table S2. Normalized fluorescence intensities and LDA output generated by **PONI-C3-Guan-Pyrene** for three bacterial species. Scores (1) and (2) correspond to the LDA plot in **Figure 2D**.

Sample Name	I/I_0				LDA output	
	Em. 378 nm	Em. 398 nm	Em. 420 nm	Em. 460 nm	Score (1)	Score (2)
S. aureus	1.350	1.307	1.480	1.672	3.643	-0.720
S. aureus	1.232	1.242	1.414	1.646	2.065	-0.771
S. aureus	1.058	1.011	1.114	1.222	2.434	0.808
S. aureus	0.985	0.943	1.010	1.078	2.127	1.054
S. aureus	0.986	0.979	1.104	1.156	2.315	0.087
S. aureus	0.934	0.881	0.897	0.982	1.449	1.845
E. coli	1.519	1.565	1.965	2.935	-2.393	0.235
E. coli	1.549	1.577	1.922	2.929	-2.744	0.834
E. coli	2.142	2.228	2.938	4.642	-4.451	-1.080
E. coli	1.564	1.685	2.172	3.473	-5.535	0.175
E. coli	1.474	1.592	1.947	3.036	-4.820	0.067
E. coli	1.005	1.106	1.272	1.733	-2.362	0.204
B. subtilis	1.612	1.632	1.985	2.565	1.854	-1.871
B. subtilis	1.575	1.607	1.885	2.471	0.991	-1.516
B. subtilis	1.253	1.230	1.480	1.971	0.518	0.854
B. subtilis	1.323	1.368	1.545	1.914	0.915	-1.144
B. subtilis	1.083	1.095	1.262	1.471	1.412	-0.080
B. subtilis	1.015	0.960	1.039	1.099	2.582	1.020

Table S3. The percentage of classification accuracy of three bacterial species based on jackknife classification (**Figure 2D**), showing an overall 61% classification accuracy.

Jackknifed Classification Matrix				
Sample Name	*B. subtilis*	*E. coli*	*S. aureus*	% correct
B. subtilis	3	0	3	50
E. coli	1	5	0	83
S. aureus	3	0	3	50
Total	7	5	6	**61**

Table S4. Normalized fluorescence intensities and LDA output generated by **Polymer-Phage** assemblies **(MOI = 0.1)** for three bacterial species. Scores (1) and (2) correspond to the LDA plot in **Figure 2E**.

Sample Name	I/I_0				LDA output	
	Em. 378 nm	Em. 398 nm	Em. 420 nm	Em. 460 nm	Score (1)	Score (2)
S. aureus	1.314	1.272	1.512	1.907	5.661	0.712
S. aureus	1.384	1.373	1.639	2.178	4.806	1.398
S. aureus	1.387	1.323	1.584	2.171	4.275	2.480
S. aureus	1.449	1.435	1.796	2.552	3.460	1.934
S. aureus	1.429	1.444	1.736	2.448	2.869	1.852
S. aureus	1.406	1.411	1.706	2.366	3.337	1.482
E. coli	1.534	1.587	2.032	3.276	-2.995	2.488
E. coli	1.537	1.574	2.052	3.272	-1.987	2.341
E. coli	1.558	1.620	2.163	3.554	-4.067	1.960
E. coli	1.537	1.543	2.088	3.450	-3.686	2.448
E. coli	1.703	1.780	2.375	4.044	-5.197	4.137
E. coli	1.424	1.462	1.853	2.952	-3.485	1.284
B. subtilis	1.004	0.931	1.143	1.402	0.540	-3.658
B. subtilis	1.024	0.989	1.211	1.550	-0.563	-3.848
B. subtilis	1.060	1.034	1.298	1.679	-0.073	-3.825
B. subtilis	0.826	0.732	0.942	1.113	-2.058	-6.310
B. subtilis	1.142	1.114	1.387	1.891	-0.227	-2.283
B. subtilis	1.067	1.077	1.379	1.810	-0.611	-4.591

Table S5. The percentage of classification accuracy of three bacterial species based on jackknife classification (**Figure 2E**), showing an overall 100% classification accuracy.

Jackknifed Classification Matrix				
**Sample Name**	***B. subtilis***	***E. coli***	***S. aureus***	**% correct**
B. subtilis	6	0	0	100
E. coli	1	6	0	100
S. aureus	3	0	6	100
**Total**	6	6	6	**100**

Table S6. Normalized fluorescence intensities and LDA output generated by **Polymer-Phage a**ssemblies **(MOI = 10)** for three bacterial species. Scores (1) and (2) correspond to the LDA plot in **Figure 2F**.

**Sample Name**	**I/I_0**				**LDA output**	
	**Em. 378 nm**	**Em. 398 nm**	**Em. 420 nm**	**Em. 460 nm**	**Score (1)**	**Score (2)**
S. aureus	1.605	1.757	2.615	4.741	-2.274	-0.101
S. aureus	1.580	1.723	2.654	4.702	-1.618	0.296
S. aureus	1.565	1.698	2.669	4.804	-1.671	0.248
S. aureus	1.654	1.807	2.706	4.937	-1.388	-0.047
S. aureus	1.574	1.666	2.518	4.541	-1.482	1.995
S. aureus	1.724	1.907	2.893	5.119	0.130	-0.516
E. coli	1.653	1.778	2.702	4.802	-0.231	1.296
E. coli	1.596	1.771	2.698	4.776	-2.040	-0.776
E. coli	1.674	1.854	2.855	5.154	-0.854	-1.029
E. coli	1.729	1.889	2.927	5.243	0.758	0.023
E. coli	1.721	1.895	2.915	5.448	-0.531	-1.144
E. coli	1.741	1.932	2.931	5.258	0.047	-0.974
B. subtilis	1.705	1.817	2.734	4.781	1.025	2.257
B. subtilis	1.874	2.051	3.159	5.603	3.260	0.185
B. subtilis	1.746	1.923	2.963	5.296	0.715	-0.490
B. subtilis	1.818	2.013	3.208	5.668	2.653	-0.889
B. subtilis	1.766	1.943	2.978	5.443	0.588	-0.707
B. subtilis	1.927	2.090	3.109	5.749	2.912	0.373

Table S7. The percentage of classification accuracy of three bacterial species based on jackknife classification (**Figure 2F**), showing an overall 50% classification accuracy.

**Jackknifed Classification Matrix**				
**Sample Name**	***B. subtilis***	***E. coli***	***S. aureus***	**% correct**
B. subtilis	3	2	1	50
E. coli	1	2	3	33
S. aureus	0	2	4	67
**Total**	4	6	8	**50**

3.2 Discrimination and identification of *B. subtilis* and three different strains of *S. aureus* (*S. aureus* ATCC 19685, MRSA CD 489, MRSA IDRL 6169)

Table S8. Normalized fluorescence intensities and LDA output generated from *B. subtilis* and three different strains of *S. aureus* incubated with **PONI-C3-Guan-Pyrene** polymer for 30 minutes. Scores (1) and (2) correspond to the LDA plot in **Figure 3B**.

Sample Name	I/I_0				LDA output	
	Em. 378 nm	Em. 398 nm	Em. 420 nm	Em. 460 nm	Score (1)	Score (2)
***S. aureus* (ATCC 19685)**	1.441	1.384	1.092	0.993	11.254	-0.175
***S. aureus* (ATCC 19685)**	1.387	1.322	1.072	0.996	8.034	-0.004
***S. aureus* (ATCC 19685)**	1.363	1.311	1.052	0.973	8.516	0.282
***S. aureus* (ATCC 19685)**	1.410	1.316	1.050	0.969	9.174	0.681
***S. aureus* (ATCC 19685)**	1.372	1.310	1.052	0.976	8.400	0.370
***S. aureus* (ATCC 19685)**	1.405	1.345	1.081	0.992	9.221	-0.124
***S. aureus* (ATCC 19685)**	1.361	1.304	1.030	0.939	9.724	0.797
***S. aureus* (ATCC 19685)**	1.390	1.338	1.047	0.982	10.111	0.560
**MRSA (CD 489)**	1.040	1.014	0.931	0.922	-4.708	1.186
**MRSA (CD 489)**	1.108	1.085	0.992	0.974	-3.685	0.163
**MRSA (CD 489)**	1.101	1.072	0.983	0.981	-4.361	0.350
**MRSA (CD 489)**	1.077	1.010	0.939	0.894	-4.368	1.258
**MRSA (CD 489)**	0.988	0.945	0.852	0.838	-4.228	2.723
**MRSA (CD 489)**	0.988	0.952	0.850	0.828	-3.493	2.761
**MRSA (CD 489)**	0.940	0.897	0.807	0.776	-4.078	3.476
**MRSA (CD 489)**	0.937	0.864	0.767	0.721	-3.358	4.427
**MRSA (IDRL 6169)**	1.211	1.186	1.046	1.029	-0.559	-0.476
**MRSA (IDRL 6169)**	1.217	1.183	1.045	1.004	-0.029	-0.406
**MRSA (IDRL 6169)**	1.250	1.202	1.057	1.036	-0.006	-0.456
**MRSA (IDRL 6169)**	1.286	1.244	1.111	1.061	0.145	-1.518
**MRSA (IDRL 6169)**	1.260	1.229	1.088	1.052	0.137	-1.151
**MRSA (IDRL 6169)**	1.349	1.298	1.152	1.115	0.841	-2.069
**MRSA (IDRL 6169)**	1.332	1.322	1.158	1.145	1.210	-2.370
**MRSA (IDRL 6169)**	1.263	1.242	1.125	1.111	-1.820	-1.987
***B. subtilis* (FD6b)**	1.040	1.026	0.963	0.954	-5.869	0.432
***B. subtilis* (FD6b)**	1.066	1.055	1.005	0.976	-6.048	-0.402
***B. subtilis* (FD6b)**	1.207	1.173	1.063	1.042	-2.226	-0.858
***B. subtilis* (FD6b)**	1.095	1.089	1.023	1.039	-6.169	-0.631
***B. subtilis* (FD6b)**	1.107	1.095	1.043	1.041	-6.416	-1.010
***B. subtilis* (FD6b)**	1.127	1.134	1.060	1.056	-4.997	-1.324
***B. subtilis* (FD6b)**	1.162	1.170	1.092	1.090	-4.712	-1.851
***B. subtilis* (FD6b)**	1.171	1.186	1.128	1.117	-5.637	-2.650

Table S9. The percentage of classification accuracy of *B. subtilis* and three different strains of *S. aureus* based on jackknife classification (**Figure 3B and 3C**), showing an overall 91% classification accuracy.

**Jackknifed Classification Matrix**					
**Sample Name**	***B. subtilis* (FD6b)**	**MRSA (CD 489)**	**MRSA (IDRL 6169)**	***S. aureus* (ATCC 19685)**	**% correct**
***B. subtilis* (FD6b)**	7	0	1	0	88
**MRSA (CD 489)**	2	6	0	0	75
**MRSA (IDRL 6169)**	0	0	8	0	100
***S. aureus* (ATCC 19685)**	0	0	0	8	100
**Total**	9	6	9	8	**91**

Table S10. Prediction of unknown bacterial cells using the training set generated from normalized fluorescence outputs in **Table S8**. The result demonstrates an overall 66% correct identification for unknown analytes (**Figure 3B and 3C**).

**Unknown sample #**	**I/I_0**				**True ID**	**Identified as**	**Correct prediction**
	**Em. 378 nm**	**Em. 398 nm**	**Em. 420 nm**	**Em. 460 nm**			
**1**	1.277	1.199	0.925	0.803	*S. aureus* (ATCC 19685)	*S. aureus* (ATCC 19685)	Yes
**2**	1.303	1.222	0.941	0.823	*S. aureus* (ATCC 19685)	*S. aureus* (ATCC 19685)	Yes
**3**	1.333	1.273	1.017	0.899	*S. aureus* (ATCC 19685)	*S. aureus* (ATCC 19685)	Yes
**4**	1.327	1.237	0.984	0.894	*S. aureus* (ATCC 19685)	*S. aureus* (ATCC 19685)	Yes
**5**	1.289	1.207	0.965	0.862	*S. aureus* (ATCC 19685)	*S. aureus* (ATCC 19685)	Yes
**6**	1.339	1.276	1.027	0.918	*S. aureus* (ATCC 19685)	*S. aureus* (ATCC 19685)	Yes
**7**	1.296	1.229	0.982	0.885	*S. aureus* (ATCC 19685)	*S. aureus* (ATCC 19685)	Yes
**8**	1.345	1.262	1.006	0.912	*S. aureus*	*S. aureus*	Yes

					(ATCC 19685)	(ATCC 19685)	
**9**	1.016	0.983	0.891	0.870	MRSA (CD 489)	MRSA (CD 489)	Yes
**10**	1.085	1.045	0.941	0.893	MRSA (CD 489)	MRSA (CD 489)	Yes
**11**	1.015	0.958	0.855	0.814	MRSA (CD 489)	MRSA (CD 489)	Yes
**12**	1.123	1.072	0.972	0.929	MRSA (CD 489)	MRSA (CD 489)	Yes
**13**	1.090	1.046	0.944	0.920	MRSA (CD 489)	MRSA (CD 489)	Yes
**14**	1.111	1.061	0.943	0.922	MRSA (CD 489)	MRSA (CD 489)	Yes
**15**	1.051	1.032	0.936	0.895	MRSA (CD 489)	MRSA (CD 489)	Yes
**16**	1.136	1.085	0.985	0.939	MRSA (CD 489)	MRSA (CD 489)	Yes
**17**	1.044	1.009	0.906	0.866	MRSA (IDRL 6169)	MRSA (CD 489)	No
**18**	1.027	0.986	0.855	0.816	MRSA (IDRL 6169)	MRSA (CD 489)	No
**19**	0.813	0.755	0.642	0.581	MRSA (IDRL 6169)	MRSA (CD 489)	No
**20**	0.931	0.864	0.761	0.688	MRSA (IDRL 6169)	MRSA (CD 489)	No
**21**	0.931	0.846	0.727	0.650	MRSA (IDRL 6169)	MRSA (CD 489)	No
**22**	0.834	0.774	0.675	0.605	MRSA (IDRL 6169)	MRSA (CD 489)	No
**23**	0.868	0.812	0.704	0.642	MRSA (IDRL 6169)	MRSA (CD 489)	No
**24**	0.932	0.865	0.741	0.679	MRSA (IDRL 6169)	MRSA (CD 489)	No
**25**	1.014	0.982	0.929	0.896	*B. subtilis* (FD6b)	*B. subtilis* (FD6b)	Yes
**26**	1.048	0.995	0.930	0.896	*B. subtilis* (FD6b)	MRSA (CD 489)	No
**27**	1.062	1.008	0.952	0.917	*B. subtilis* (FD6b)	MRSA (CD 489)	No
**28**	1.134	1.098	1.033	1.010	*B. subtilis* (FD6b)	*B. subtilis* (FD6b)	Yes
**29**	1.102	1.063	0.988	0.955	*B. subtilis* (FD6b)	*B. subtilis* (FD6b)	Yes
**30**	1.180	1.151	1.090	1.060	*B. subtilis* (FD6b)	*B. subtilis* (FD6b)	Yes
**31**	1.067	1.027	0.971	0.928	*B. subtilis* (FD6b)	*B. subtilis* (FD6b)	Yes
**32**	1.021	0.971	0.896	0.856	*B. subtilis* (FD6b)	MRSA (CD 489)	No

Table S11. Normalized fluorescence intensities and LDA output generated from *B. subtilis* and three different strains of *S. aureus* incubated with **Polymer-Phage** assemblies **(MOI = 0.1)** for 30 min. Scores (1) and (2) correspond to the LDA plot in **Figure 3E**.

**Sample Name**	**I/I_0**				**LDA output**	
	**Em. 378 nm**	**Em. 398 nm**	**Em. 420 nm**	**Em. 460 nm**	**Score (1)**	**Score (2)**
***S. aureus* (ATCC 19685)**	1.351	1.291	1.017	0.922	-10.669	0.170
***S. aureus* (ATCC 19685)**	1.377	1.292	1.046	0.953	-8.301	-0.267
***S. aureus* (ATCC 19685)**	1.361	1.275	1.026	0.918	-9.046	-1.219
***S. aureus* (ATCC 19685)**	1.439	1.354	1.052	0.942	-12.453	-0.170
***S. aureus* (ATCC 19685)**	1.463	1.362	1.047	0.920	-13.596	-0.851
***S. aureus* (ATCC 19685)**	1.448	1.376	1.111	1.015	-10.069	-0.657
***S. aureus* (ATCC 19685)**	1.405	1.331	1.071	0.983	-9.370	0.178
***S. aureus* (ATCC 19685)**	1.356	1.280	0.978	0.888	-11.685	1.893
**MRSA (CD 489)**	1.060	1.010	0.925	0.909	4.141	1.978
**MRSA (CD 489)**	1.152	1.101	1.004	0.995	3.546	2.300
**MRSA (CD 489)**	1.249	1.180	1.084	1.055	3.003	0.401
**MRSA (CD 489)**	1.211	1.134	1.045	1.018	3.748	0.817
**MRSA (CD 489)**	1.204	1.171	1.089	1.069	3.388	0.061
**MRSA (CD 489)**	1.191	1.153	1.062	1.062	3.817	2.117
**MRSA (CD 489)**	1.097	1.069	0.978	0.973	3.590	2.249
**MRSA (CD 489)**	1.088	1.027	0.926	0.905	3.223	2.191
**MRSA (IDRL 6169)**	1.252	1.181	1.060	1.032	1.566	1.379
**MRSA (IDRL 6169)**	1.205	1.135	1.028	0.982	1.848	-0.284
**MRSA (IDRL 6169)**	1.213	1.138	1.030	0.992	2.137	0.437
**MRSA (IDRL 6169)**	1.276	1.220	1.100	1.056	0.578	-0.597
**MRSA (IDRL 6169)**	1.265	1.184	1.072	1.040	2.199	0.862
**MRSA (IDRL 6169)**	1.232	1.174	1.079	1.024	1.849	-2.001
**MRSA (IDRL 6169)**	1.265	1.214	1.117	1.066	1.643	-1.956
**MRSA (IDRL 6169)**	1.223	1.179	1.077	1.049	2.056	0.220
***B. subtilis* (FD6b)**	1.158	1.113	1.052	1.026	4.782	-0.553
***B. subtilis* (FD6b)**	1.119	1.076	1.026	1.005	5.678	-0.232
***B. subtilis* (FD6b)**	1.165	1.123	1.062	1.036	4.725	-0.749
***B. subtilis* (FD6b)**	1.199	1.155	1.110	1.090	5.931	-0.966
***B. subtilis* (FD6b)**	1.203	1.157	1.103	1.064	4.670	-2.099
***B. subtilis* (FD6b)**	1.187	1.147	1.110	1.085	6.045	-1.674
***B. subtilis* (FD6b)**	1.209	1.191	1.156	1.130	5.548	-2.541
***B. subtilis* (FD6b)**	1.215	1.163	1.107	1.088	5.476	-0.437

Table S12. The percentage of classification accuracy of *B. subtilis* and three different strains of *S. aureus* based on jackknife classification (**Figure 3E and 3F**), showing an overall 94% classification accuracy.

**Jackknifed Classification Matrix**					
**Sample Name**	***B. subtilis* (FD6b)**	**MRSA (CD 489)**	**MRSA (IDRL 6169)**	***S. aureus* (ATCC 19685)**	**% correct**
***B. subtilis* (FD6b)**	8	0	0	0	100
**MRSA (CD 489)**	0	6	2	0	75
**MRSA (IDRL 6169)**	0	0	8	0	100
***S. aureus* (ATCC 19685)**	0	0	0	8	100
**Total**	8	6	10	8	**94**

Table S13. Prediction of unknown bacterial cells using the training set generated from normalized fluorescence outputs in **Table S11**. The result demonstrates an overall 100% correct identification for unknown analytes (**Figure 3E** and **3F**).

**Unknown sample #**	**I/I_0**				**True ID**	**Identified as**	**Correct prediction**
	**Em. 378 nm**	**Em. 398 nm**	**Em. 420 nm**	**Em. 460 nm**			
**1**	1.288	1.210	0.979	0.873	*S. aureus* (ATCC 19685)	*S. aureus* (ATCC 19685)	Yes
**2**	1.291	1.199	0.970	0.859	*S. aureus* (ATCC 19685)	*S. aureus* (ATCC 19685)	Yes
**3**	1.302	1.197	0.953	0.860	*S. aureus* (ATCC 19685)	*S. aureus* (ATCC 19685)	Yes
**4**	1.326	1.232	0.991	0.885	*S. aureus* (ATCC 19685)	*S. aureus* (ATCC 19685)	Yes
**5**	1.312	1.242	0.987	0.894	*S. aureus* (ATCC 19685)	*S. aureus* (ATCC 19685)	Yes
**6**	1.267	1.195	0.958	0.877	*S. aureus* (ATCC 19685)	*S. aureus* (ATCC 19685)	Yes
**7**	1.270	1.200	0.967	0.879	*S. aureus* (ATCC 19685)	*S. aureus* (ATCC 19685)	Yes
**8**	1.304	1.216	0.985	0.883	*S. aureus* (ATCC 19685)	*S. aureus* (ATCC 19685)	Yes
**9**	1.084	1.005	0.904	0.867	MRSA (CD 489)	MRSA (CD 489)	Yes
**10**	1.077	0.999	0.914	0.872	MRSA (CD	MRSA (CD	Yes

					489)	489)	
**11**	1.076	1.004	0.914	0.872	MRSA (CD 489)	MRSA (CD 489)	Yes
**12**	1.117	1.025	0.929	0.886	MRSA (CD 489)	MRSA (CD 489)	Yes
**13**	1.058	0.988	0.895	0.865	MRSA (CD 489)	MRSA (CD 489)	Yes
**14**	1.139	1.075	0.983	0.946	MRSA (CD 489)	MRSA (CD 489)	Yes
**15**	1.048	0.989	0.900	0.865	MRSA (CD 489)	MRSA (CD 489)	Yes
**16**	1.086	1.008	0.939	0.911	MRSA (CD 489)	MRSA (CD 489)	Yes
**17**	1.201	1.125	1.013	0.975	MRSA (IDRL 6169)	MRSA (IDRL 6169)	Yes
**18**	1.200	1.128	1.022	0.974	MRSA (IDRL 6169)	MRSA (IDRL 6169)	Yes
**19**	1.206	1.105	0.993	0.946	MRSA (IDRL 6169)	MRSA (IDRL 6169)	Yes
**20**	1.174	1.090	0.983	0.936	MRSA (IDRL 6169)	MRSA (IDRL 6169)	Yes
**21**	1.180	1.095	0.988	0.940	MRSA (IDRL 6169)	MRSA (IDRL 6169)	Yes
**22**	1.176	1.116	1.031	0.973	MRSA (IDRL 6169)	MRSA (IDRL 6169)	Yes
**23**	1.148	1.088	0.982	0.930	MRSA (IDRL 6169)	MRSA (IDRL 6169)	Yes
**24**	1.113	1.055	0.958	0.906	MRSA (IDRL 6169)	MRSA (IDRL 6169)	Yes
**25**	1.063	1.020	0.964	0.935	*B. subtilis* (FD6b)	*B. subtilis* (FD6b)	Yes
**26**	1.116	1.058	1.009	0.977	*B. subtilis* (FD6b)	*B. subtilis* (FD6b)	Yes
**27**	1.149	1.077	1.034	0.994	*B. subtilis* (FD6b)	*B. subtilis* (FD6b)	Yes
**28**	1.139	1.091	1.044	1.008	*B. subtilis* (FD6b)	*B. subtilis* (FD6b)	Yes
**29**	1.099	1.063	1.000	0.973	*B. subtilis* (FD6b)	*B. subtilis* (FD6b)	Yes
**30**	1.153	1.121	1.068	1.045	*B. subtilis* (FD6b)	*B. subtilis* (FD6b)	Yes
**31**	1.139	1.114	1.070	1.051	*B. subtilis* (FD6b)	*B. subtilis* (FD6b)	Yes
**32**	1.102	1.069	1.026	1.004	*B. subtilis* (FD6b)	*B. subtilis* (FD6b)	Yes

Table S14. Normalized fluorescence intensities and LDA output generated from *B. subtilis* and three different strains of *S. aureus* incubated with **PONI-C3-Guan-Pyrene** polymer for 1 hour (**Figure 3C**).

Sample Name	I/I_0				LDA output	
	**Em. 378 nm**	**Em. 398 nm**	**Em. 420 nm**	**Em. 460 nm**	**Score (1)**	**Score (2)**
S. aureus **(ATCC 19685)**	1.377	1.299	0.978	0.851	12.433	0.665
S. aureus **(ATCC 19685)**	1.440	1.341	1.011	0.865	14.072	-0.028
S. aureus **(ATCC 19685)**	1.457	1.368	1.071	0.949	11.444	-1.067
S. aureus **(ATCC 19685)**	1.439	1.346	1.060	0.924	11.076	-1.257
S. aureus **(ATCC 19685)**	1.396	1.327	1.026	0.918	10.763	-0.105
S. aureus **(ATCC 19685)**	1.467	1.383	1.099	0.983	10.515	-1.615
S. aureus **(ATCC 19685)**	1.403	1.339	1.027	0.935	11.050	0.293
S. aureus **(ATCC 19685)**	1.439	1.363	1.067	0.950	10.990	-0.978
**MRSA (CD 489)**	1.076	1.044	0.945	0.901	-4.946	-0.444
**MRSA (CD 489)**	1.180	1.107	0.982	0.929	-2.000	-0.788
**MRSA (CD 489)**	1.086	1.025	0.890	0.832	-1.925	0.774
**MRSA (CD 489)**	1.193	1.129	1.002	0.942	-1.807	-1.203
**MRSA (CD 489)**	1.178	1.132	1.002	0.938	-1.918	-1.252
**MRSA (CD 489)**	1.183	1.117	1.001	0.950	-2.715	-1.219
**MRSA (CD 489)**	1.129	1.098	0.965	0.939	-3.024	-0.037
**MRSA (CD 489)**	1.200	1.148	1.003	0.960	-1.264	-0.730
**MRSA (IDRL 6169)**	1.112	1.080	0.934	0.895	-2.034	0.445
**MRSA (IDRL 6169)**	1.089	1.056	0.891	0.829	-0.461	1.045
**MRSA (IDRL 6169)**	0.885	0.800	0.680	0.601	-3.019	3.705
**MRSA (IDRL 6169)**	0.991	0.917	0.794	0.723	-2.697	1.967
**MRSA (IDRL 6169)**	0.948	0.888	0.735	0.675	-1.529	3.483
**MRSA (IDRL 6169)**	0.902	0.816	0.702	0.626	-3.382	3.283
**MRSA (IDRL 6169)**	0.903	0.861	0.719	0.666	-2.857	3.684
**MRSA (IDRL 6169)**	0.983	0.918	0.767	0.703	-1.335	2.862
B. subtilis **(FD6b)**	1.039	1.007	0.937	0.913	-7.325	-0.371
B. subtilis **(FD6b)**	1.069	1.016	0.945	0.908	-6.498	-0.643
B. subtilis **(FD6b)**	1.074	1.033	0.973	0.940	-7.394	-1.213
B. subtilis **(FD6b)**	1.142	1.119	1.059	1.029	-7.386	-2.599
B. subtilis **(FD6b)**	1.135	1.067	1.006	0.955	-6.239	-1.981
B. subtilis **(FD6b)**	1.199	1.156	1.106	1.084	-7.602	-3.363
B. subtilis **(FD6b)**	1.077	1.054	0.973	0.935	-6.274	-1.015
B. subtilis **(FD6b)**	1.019	0.981	0.910	0.861	-6.713	-0.294

Table S15. The percentage of classification accuracy of *B. subtilis* and three different strains of *S. aureus* based on jackknife classification (**Figure 3C**), showing an overall 91% classification accuracy.

**Jackknifed Classification Matrix**					
**Sample Name**	***B. subtilis* (FD6b)**	**MRSA (CD 489)**	**MRSA (IDRL 6169)**	***S. aureus* (ATCC 19685)**	**% correct**
***B. subtilis* (FD6b)**	7	0	1	0	88
**MRSA (CD 489)**	2	6	0	0	75
**MRSA (IDRL 6169)**	0	0	8	0	100
***S. aureus* (ATCC 19685)**	0	0	0	8	100
**Total**	9	6	9	8	**91**

Table S16. Prediction of unknown bacterial cells using the training set generated from normalized fluorescence outputs in **Table S14**. The result demonstrates an overall 63% correct identification for unknown analytes (**Figure 3F**).

**Unknown sample #**	**I/I_0**				**True ID**	**Identified as**	**Correct prediction**
	**Em. 378 nm**	**Em. 398 nm**	**Em. 420 nm**	**Em. 460 nm**			
**1**	1.568	1.503	1.138	1.030	*S. aureus* (ATCC 19685)	*S. aureus* (ATCC 19685)	Yes
**2**	1.487	1.429	1.111	1.017	*S. aureus* (ATCC 19685)	*S. aureus* (ATCC 19685)	Yes
**3**	1.484	1.428	1.093	0.992	*S. aureus* (ATCC 19685)	MRSA (CD 489)	No
**4**	1.503	1.422	1.110	0.990	*S. aureus* (ATCC 19685)	*S. aureus* (ATCC 19685)	Yes
**5**	1.509	1.434	1.115	1.002	*S. aureus* (ATCC 19685)	*S. aureus* (ATCC 19685)	Yes
**6**	1.498	1.439	1.120	0.998	*S. aureus* (ATCC 19685)	*S. aureus* (ATCC 19685)	Yes
**7**	1.440	1.372	1.069	0.963	*S. aureus* (ATCC 19685)	*S. aureus* (ATCC 19685)	Yes
**8**	1.483	1.417	1.106	0.996	*S. aureus* (ATCC 19685)	*S. aureus* (ATCC 19685)	Yes
**9**	1.086	1.055	0.962	0.935	MRSA (CD 489)	*S. aureus* (ATCC 19685)	No
**10**	1.153	1.114	1.009	0.992	MRSA (CD 489)	MRSA (CD 489)	Yes
**11**	1.152	1.111	1.009	1.001	MRSA (CD 489)	MRSA (CD 489)	Yes
**12**	1.115	1.042	0.950	0.909	MRSA (CD 489)	MRSA (CD 489)	Yes
**13**	1.031	0.980	0.888	0.853	MRSA (CD 489)	*S. aureus* (ATCC 19685)	No

**14**	1.035	0.984	0.887	0.846	MRSA (CD 489)	MRSA (CD 489)	Yes
**15**	0.973	0.930	0.819	0.777	MRSA (CD 489)	MRSA (IDRL 6169	No
**16**	0.964	0.895	0.793	0.737	MRSA (CD 489)	MRSA (IDRL 6169	No
**17**	1.275	1.257	1.079	1.048	MRSA (IDRL 6169	MRSA (CD 489)	No
**18**	1.285	1.246	1.080	1.014	MRSA (IDRL 6169	MRSA (CD 489)	No
**19**	1.302	1.283	1.069	1.046	MRSA (IDRL 6169	MRSA (CD 489)	No
**20**	1.331	1.309	1.132	1.090	MRSA (IDRL 6169	MRSA (CD 489)	No
**21**	1.315	1.296	1.121	1.065	MRSA (IDRL 6169	MRSA (CD 489)	No
**22**	1.394	1.370	1.182	1.135	MRSA (IDRL 6169	MRSA (CD 489)	No
**23**	1.382	1.392	1.185	1.152	MRSA (IDRL 6169	MRSA (CD 489)	No
**24**	1.316	1.291	1.143	1.118	MRSA (IDRL 6169	MRSA (CD 489)	No
**25**	1.062	1.051	0.985	0.965	*B. subtilis* (FD6b)	*B. subtilis* (FD6b)	Yes
**26**	1.076	1.056	1.005	0.980	*B. subtilis* (FD6b)	*B. subtilis* (FD6b)	Yes
**27**	1.228	1.180	1.070	1.037	*B. subtilis* (FD6b)	*B. subtilis* (FD6b)	Yes
**28**	1.100	1.097	1.023	1.020	*B. subtilis* (FD6b)	*B. subtilis* (FD6b)	Yes
**29**	1.119	1.114	1.059	1.041	*B. subtilis* (FD6b)	*B. subtilis* (FD6b)	Yes
**30**	1.136	1.128	1.058	1.045	*B. subtilis* (FD6b)	*B. subtilis* (FD6b)	Yes
**31**	1.141	1.140	1.072	1.057	*B. subtilis* (FD6b)	*B. subtilis* (FD6b)	Yes
**32**	1.165	1.185	1.113	1.104	*B. subtilis* (FD6b)	*B. subtilis* (FD6b)	Yes

Table S17. Normalized fluorescence intensities and LDA output generated from *B. subtilis* and three different strains of *S. aureus* incubated with **Polymer-Phage** assemblies **(MOI = 0.1)** for 60 min (**Figure 3F**).

Sample Name	I/I_0				LDA output	
	Em. 378 nm	Em. 398 nm	Em. 420 nm	Em. 460 nm	Score (1)	Score (2)
***S. aureus* (ATCC 19685)**	1.493	1.442	1.120	1.018	7.833	-1.953
***S. aureus* (ATCC 19685)**	1.447	1.362	1.021	0.895	9.772	-0.229
***S. aureus* (ATCC 19685)**	1.321	1.233	0.946	0.843	7.395	1.378
***S. aureus* (ATCC 19685)**	1.691	1.582	1.229	1.094	9.197	-2.999
***S. aureus* (ATCC 19685)**	1.431	1.325	1.048	0.928	6.594	-0.344
***S. aureus* (ATCC 19685)**	1.397	1.288	1.019	0.911	6.247	0.521
***S. aureus* (ATCC 19685)**	1.364	1.278	1.009	0.909	6.163	0.324
***S. aureus* (ATCC 19685)**	1.377	1.298	1.027	0.923	6.162	-0.174
**MRSA (CD 489)**	1.082	0.997	0.872	0.817	-0.282	2.963
**MRSA (CD 489)**	1.172	1.089	0.965	0.898	-0.755	1.160
**MRSA (CD 489)**	1.159	1.091	0.952	0.902	-0.225	1.616
**MRSA (CD 489)**	1.191	1.076	0.954	0.906	-0.974	2.718
**MRSA (CD 489)**	1.140	1.073	0.952	0.897	-1.027	1.302
**MRSA (CD 489)**	1.215	1.136	1.037	0.977	-2.469	-0.007
**MRSA (CD 489)**	1.094	1.039	0.931	0.905	-1.918	2.187
**MRSA (CD 489)**	1.078	1.019	0.921	0.881	-2.055	1.931
**MRSA (IDRL 6169)**	1.324	1.271	1.114	1.074	-0.598	-0.773
**MRSA (IDRL 6169)**	1.284	1.224	1.110	1.076	-2.655	-0.616
**MRSA (IDRL 6169)**	1.241	1.174	1.013	0.943	0.693	0.143
**MRSA (IDRL 6169)**	1.266	1.168	1.034	0.973	-0.765	0.693
**MRSA (IDRL 6169)**	1.300	1.218	1.082	1.003	-0.718	-0.973
**MRSA (IDRL 6169)**	1.279	1.220	1.067	1.010	-0.247	-0.496
**MRSA (IDRL 6169)**	1.253	1.172	1.030	0.961	-0.298	0.192
**MRSA (IDRL 6169)**	1.267	1.201	1.084	1.018	-1.830	-1.112
***B. subtilis* (FD6b)**	1.153	1.164	1.089	1.095	-5.007	-1.013
***B. subtilis* (FD6b)**	1.205	1.212	1.154	1.152	-6.056	-2.277
***B. subtilis* (FD6b)**	1.153	1.094	1.048	1.009	-5.287	-0.308
***B. subtilis* (FD6b)**	1.174	1.102	1.045	1.009	-4.802	0.266
***B. subtilis* (FD6b)**	1.183	1.124	1.069	1.029	-5.005	-0.603
***B. subtilis* (FD6b)**	1.178	1.129	1.077	1.054	-5.450	-0.419
***B. subtilis* (FD6b)**	1.153	1.145	1.103	1.080	-6.126	-1.940
***B. subtilis* (FD6b)**	1.123	1.101	1.056	1.023	-5.505	-1.157

Table S18. The percentage of classification accuracy of *B. subtilis* and three different strains of *S. aureus* based on jackknife classification (**Figure 3F**), showing an overall 97% classification accuracy.

**Jackknifed Classification Matrix**					
**Sample Name**	***B. subtilis* (FD6b)**	**MRSA (CD 489)**	**MRSA (IDRL 6169)**	***S. aureus* (ATCC 19685)**	**% correct**
***B. subtilis* (FD6b)**	8	0	0	0	100
**MRSA (CD 489)**	0	7	1	0	88
**MRSA (IDRL 6169)**	0	0	8	0	100
***S. aureus* (ATCC 19685)**	0	0	0	8	100
**Total**	9	9	6	8	**97**

Table S19. Prediction of unknown bacterial cells using the training set from generated from normalized fluorescence outputs in **Table S17**. The result demonstrates an overall 94% correct identification for unknown analytes (**Figure 3F**).

**Unknown sample #**	**I/I_0**				**True ID**	**Identified as**	**Correct prediction**
	**Em. 378 nm**	**Em. 398 nm**	**Em. 420 nm**	**Em. 460 nm**			
**1**	1.493	1.414	1.110	0.993	*S. aureus* (ATCC 19685)	*S. aureus* (ATCC 19685)	Yes
**2**	1.551	1.477	1.144	1.038	*S. aureus* (ATCC 19685)	*S. aureus* (ATCC 19685)	Yes
**3**	1.547	1.452	1.093	0.964	*S. aureus* (ATCC 19685)	*S. aureus* (ATCC 19685)	Yes
**4**	1.565	1.474	1.098	0.951	*S. aureus* (ATCC 19685)	*S. aureus* (ATCC 19685)	Yes
**5**	1.518	1.426	1.050	0.926	*S. aureus* (ATCC 19685)	*S. aureus* (ATCC 19685)	Yes
**6**	1.548	1.451	1.087	0.935	*S. aureus* (ATCC 19685)	*S. aureus* (ATCC 19685)	Yes
**7**	1.419	1.327	1.051	0.934	*S. aureus* (ATCC 19685)	*S. aureus* (ATCC 19685)	Yes
**8**	1.363	1.283	1.016	0.926	*S. aureus* (ATCC 19685)	*S. aureus* (ATCC 19685)	Yes
**9**	1.090	1.005	0.851	0.795	MRSA (CD 489)	MRSA (CD 489)	Yes
**10**	1.156	1.088	0.961	0.896	MRSA (CD 489)	MRSA (CD 489)	Yes
**11**	1.110	1.054	0.976	0.931	MRSA (CD	MRSA (CD	Yes

					489)	489)	
**12**	1.251	1.191	1.078	1.038	MRSA (CD 489)	MRSA (IDRL 6169	No
**13**	1.254	1.216	1.087	1.061	MRSA (CD 489)	MRSA (IDRL 6169	No
**14**	1.108	1.064	0.939	0.904	MRSA (CD 489)	MRSA (CD 489)	Yes
**15**	1.111	1.043	0.945	0.896	MRSA (CD 489)	MRSA (CD 489)	Yes
**16**	1.137	1.063	0.964	0.928	MRSA (CD 489)	MRSA (CD 489)	Yes
**17**	1.291	1.220	1.069	1.022	MRSA (IDRL 6169	MRSA (IDRL 6169	Yes
**18**	1.330	1.272	1.136	1.087	MRSA (IDRL 6169	MRSA (IDRL 6169	Yes
**19**	1.316	1.244	1.105	1.039	MRSA (IDRL 6169	MRSA (IDRL 6169	Yes
**20**	1.286	1.208	1.048	1.000	MRSA (IDRL 6169	MRSA (IDRL 6169	Yes
**21**	1.336	1.273	1.118	1.064	MRSA (IDRL 6169	MRSA (IDRL 6169	Yes
**22**	1.303	1.229	1.105	1.046	MRSA (IDRL 6169	MRSA (IDRL 6169	Yes
**23**	1.243	1.178	1.039	0.973	MRSA (IDRL 6169	MRSA (IDRL 6169	Yes
**24**	1.275	1.186	1.058	0.991	MRSA (IDRL 6169	MRSA (IDRL 6169	Yes
**25**	1.243	1.209	1.152	1.142	*B. subtilis* (FD6b)	*B. subtilis* (FD6b)	Yes
**26**	1.206	1.179	1.113	1.091	*B. subtilis* (FD6b)	*B. subtilis* (FD6b)	Yes
**27**	1.267	1.230	1.182	1.156	*B. subtilis* (FD6b)	*B. subtilis* (FD6b)	Yes
**28**	1.236	1.192	1.118	1.097	*B. subtilis* (FD6b)	*B. subtilis* (FD6b)	Yes
**29**	1.222	1.188	1.118	1.094	*B. subtilis* (FD6b)	*B. subtilis* (FD6b)	Yes
**30**	1.203	1.168	1.124	1.102	*B. subtilis* (FD6b)	*B. subtilis* (FD6b)	Yes
**31**	1.136	1.084	1.043	1.004	*B. subtilis* (FD6b)	*B. subtilis* (FD6b)	Yes
**32**	1.194	1.142	1.104	1.086	*B. subtilis* (FD6b)	*B. subtilis* (FD6b)	Yes

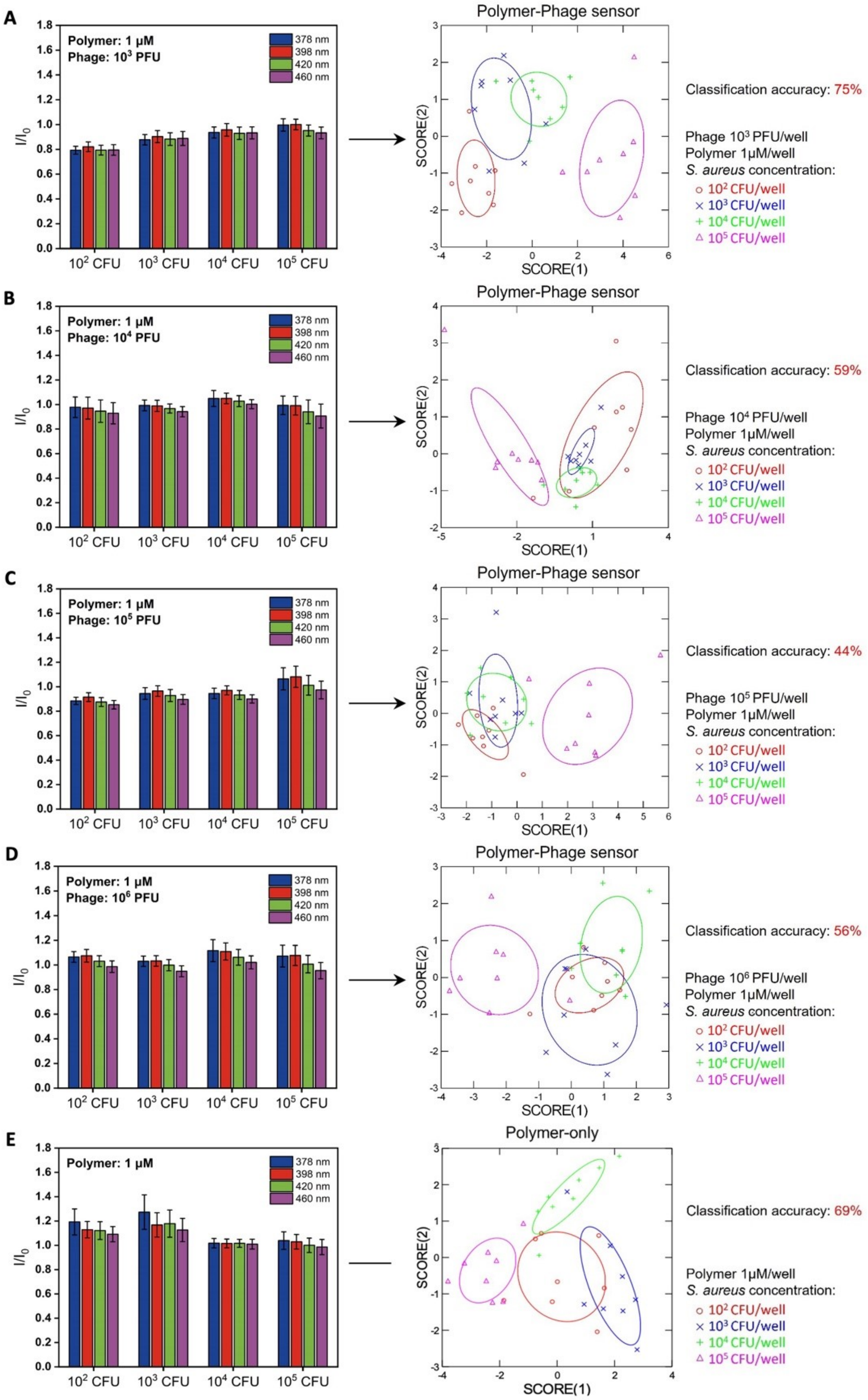

3.3 Discrimination of bacterial concentrations

Figure S7. Differentiation of bacterial concentrations using the polymer-phage sensor after 1 h of incubation. On the left side, fluorescence signal from four channels normalized to the sensor only (I/I_0). On the right side, LDA of the first two canonical score plots of the fluorescence response patterns. All the experiments included eight biological repetitions.

3.4 Overview of phage-based biosensors and commercial diagnostic platforms

Table S20. Comparison of phage-based biosensing studies and commercial diagnostic platforms across key performance parameters.

Sensor	Species detection	Strain detection	LOD	Range of detection	Detection time	Ref
**Array-based polymer-phage fluorescent sensor with LDA**	*S. aureus, E. coli, B. subtilis*	Differentiates MRSA vs MSSA:<br><br>Clinical isolates *S. aureus* ATCC 19685, MRSA CD 489, MRSA IDRL 6169	~100 CFU* without amplification	10^2-10^7 CFU*<br><br>10^6 CFU**<br><br>10^7 CFU ***	1 - 1,5 h total (30 min for sensor preparation + 30 min ** 60 min *** + LDA)<br><br>* - *S. aureus* ATCC 19685<br>** - species detection and differentiation<br>*** - strain detection and differentiation	
**Cocktail of dual-modified M13 phages with flow cytometry**	*E. coli* O157:H7, *S. Typhimurium, P. aeruginosa*	*NA – species-level only*	~10^2 cells/mL	10^2 - 10^6 CFU/mL	~1.5 - 2 h total (30 min phage incubation + 60 min labeling + data acquisition)	[2]
**Phage-based magnetic relaxation switching (MRS) biosensor**	*S. Typhimurium, S. Paratyphi B, S. Enteritidis*	*NA – species-level only*	5 CFU/mL (with 4 h pre-enrichment) ; 10^3 CFU/mL without amplification	10^2 - 10^8 CFU/mL	~5 h total (4 h pre-enrichment + ~1 h biosensor assay)	[3]
**Dual-mode hydrogel array with phage-DNA probe**	*E. coli* O157:H7<br><br>*S. Typhimurium*	*NA – species-level only*	50 CFU/mL (FL); 6 CFU/mL (MCE) for *E. coli* O157:H7	10–10^6 CFU/mL	~45–60 min total (15 min phage binding + 30 min RCA + 10 s FL + MCE)	[4]
**Signal-enhanced magnetoelastic biosensor with humidity-resistant phages**	*E. coli O157:H7*<br><br>*S. Typhimurium*	*NA – species-level only*	1.7 log CFU/25 mm^2 (*S. Typhimurium*), 1.6 log CFU/25 mm^2 (*E. coli*)	2–8 log CFU/25 mm^2	16 min (phage binding only; no enrichment or prep)	[5]

**Phage T156-AuNP visual colorimetric biosensor**	*(NA – only S. Typhimurium)*	3 strains of *S. Typhimurium*: ATCC 13311, 14028, 13076;	38 CFU/mL	3.8×10^1 to 3.8×10^9 CFU/mL	~80 min	[6]
**Phage T156-based amplification assay (PAA) + qPCR**	*(NA – only S. Typhimurium)*	5 strains of *S. Typhimurium*: ATCC 13311, 14028, 13076; SJTUF 13277, 13350	1 CFU/mL (plaque assay); 10 CFU/mL (PAA–qPCR)	10^0–10^8 CFU/mL	3.5 h (qPCR); 6.5 h (plaque-based)	[7]
**Photoacoustic cytometry with dyed SP1 phage**	*(NA – only S. aureus)*	4 strains of *S. aureus*	Not explicitly reported; inferred single-cell sensitivity	2 - 15 cells per 0.04 µL detection volume	<4 h total (2 h antibiotic exposure + ~2 h photoacoustic scan)	[8]
**Photoacoustic cytometry with dyed SP1 phage**	*(NA – only S. aureus*)	13 strains of *S. aureus*	Not explicitly reported	2 - 818 cells per sample	~4 h total (2 h antibiotic treatment + 90 - 120 min photoacoustic scan)	[9]
**BioFire® FilmArray Gastrointestinal Panel (PCR panel)**	Salmonella spp., *E. coli* (O157, EPEC, EAEC, etc. 22 targets)	Limited to gene markers	10^3–10^4 CFU/mL	Qualitative – per gene	~1 h	bioMérieux, BioFire Diagnostics
**Illumina WGS + DRAGEN pipeline**	All known bacterial species	Full strain-level typing, AMR, MLST, SNP tree	NA (requires isolated DNA)	Genome-wide	24 – 48 h	Illumina Inc
**Bruker MALDI Biotyper® (MALDI-TOF MS)**	480 bacterial species	*NA – species-level only*	Single colony	Requires pure colonies	<10 min	Bruker Daltonics
**SSI Diagnostica Sero-Quick**	*Salmonella enterica* (all major serovars)	Serovar-level (strain-type for regulated pathogens)	Not specified	Serovar-specific	~2–4 h	SSI Diagnostica, Denmark
**Mammoth DETECTR™**	Programmable *E. coli, Salmonella*, etc.	SNP-level resolution	~10 copies/µL	Depends on assay	~30 min	Mammoth Biosciences

Reference

1. Landis, R. F. *et al.* Cross-Linked Polymer-Stabilized Nanocomposites for the Treatment of Bacterial Biofilms. *ACS Nano* **11**, 946–952 (2017).

2. Wu, L. *et al.* Multiplexed detection of bacterial pathogens based on a cocktail of dual-modified phages. *Anal. Chim. Acta* **1166**, 338596 (2021).

3. Huang, C. *et al.* A phage-based magnetic relaxation switching biosensor using bioorthogonal reaction signal amplification for Salmonella detection in foods. *Food Chem.* **400**, 134035 (2023).

4. Xu, J. *et al.* A universal dual-mode hydrogel array based on phage-DNA probe for simultaneous rapid screening and precisely quantitative detection of Escherichia coli O157:H7 in foods by the fluorescent/microfluidic chip electrophoresis methods. *Anal. Chim. Acta* **1287**, 342053 (2024).

5. Choi, I. Y., Choe, J., Chin, B. A. & Park, M. K. User-friendly, signal-enhanced planar spiral coil-based magnetoelastic biosensor combined with humidity-resistant phages for simultaneous detection of Salmonella Typhimurium and Escherichia coli O157:H7 on fresh produce. *Sensors Actuators B Chem.* **393**, 134179 (2023).

6. Wang, Y. *et al.* A visual colorimetric assay based on phage T156 and gold nanoparticles for the sensitive detection of Salmonella in lettuce. *Anal. Chim. Acta* **1272**, 341501 (2023).

7. Huang, C. *et al.* Phage amplification-based technologies for simultaneous quantification of viable Salmonella in foodstuff and rapid antibiotic susceptibility testing. *Food Res. Int.* **156**, 111279 (2022).

8. Edgar, R. H. *et al.* Photoacoustic discrimination of antibiotic-resistant and sensitive Staphylococcus aureus isolates. *Lasers Surg. Med.* **54**, 418–425 (2022).

9. Edgar, R. H. *et al.* Differentiating methicillin-resistant and susceptible Staphylococcus aureus from ocular infections using photoacoustic labeling. *Front. Med.* **10**, 1–8 (2023).
